# Functional Modulation of Vascular Adhesion Protein-1 by a Novel Splice Variant

**DOI:** 10.1371/journal.pone.0054151

**Published:** 2013-01-18

**Authors:** Sam Kaitaniemi, Kirsi Grön, Heli Elovaara, Marko Salmi, Sirpa Jalkanen, Kati Elima

**Affiliations:** 1 MediCity Research Laboratory, University of Turku, Turku, Finland; 2 Department of Microbiology and Immunology, University of Turku, Turku, Finland; 3 Department of Medical Biochemistry and Genetics, University of Turku, Turku, Finland; University of Medicine and Dentistry of New Jersey, United States of America

## Abstract

Vascular Adhesion Protein-1 (VAP-1) is an endothelial adhesion molecule belonging to the primary amine oxidases. Upon inflammation it takes part in the leukocyte extravasation cascade facilitating transmigration of leukocytes into the inflamed tissue. Screening of a human lung cDNA library revealed the presence of an alternatively spliced shorter transcript of VAP-1, VAP-1Δ3. Here, we have studied the functional and structural characteristics of VAP-1Δ3, and show that the mRNA for this splice variant is expressed in most human tissues studied. In comparison to the parent molecule this carboxy-terminally truncated isoform lacks several of the amino acids important in the formation of the enzymatic groove of VAP-1. In addition, the conserved His684, which takes part in coordinating the active site copper, is missing from VAP-1Δ3. Assays using the prototypic amine substrates methylamine and benzylamine demonstrated that VAP-1Δ3 is indeed devoid of the semicarbazide-sensitive amine oxidase (SSAO) activity characteristic to VAP-1. When VAP-1Δ3-cDNA is transfected into cells stably expressing VAP-1, the surface expression of the full-length molecule is reduced. Furthermore, the SSAO activity of the co-transfectants is diminished in comparison to transfectants expressing only VAP-1. The observed down-regulation of both the expression and enzymatic activity of VAP-1 may result from a dominant-negative effect caused by heterodimerization between VAP-1 and VAP-1Δ3, which was detected in co-immunoprecipitation studies. This alternatively spliced transcript adds thus to the repertoire of potential regulatory mechanisms through which the cell-surface expression and enzymatic activity of VAP-1 can be modulated.

## Introduction

The proper functioning of the immune system relies on the continuous recirculation of lymphocytes throughout the body and the ability of leukocytes to migrate to the sites of inflammation. The extravasation of leukocytes from blood to tissues is brought about by several adhesion molecules on the trafficking cells, which recognize their counter-receptors on vascular endothelium [1], [2]. One of these endothelial adhesion molecules is Vascular Adhesion Protein-1 (VAP-1), a 170-kDa homodimeric transmembrane glycoprotein, which is heavily sialylated [3], [4]. VAP-1 is expressed constitutively on high endothelial venules (HEVs) of lymphoid tissues, and in flat-walled venules of the lamina propria in the gut [5]. It is also expressed on adipocytes, smooth muscle cells, pericytes, and follicular dendritic cells of germinal centers [3], [5]–[7]. The cell-surface expression of VAP-1 is increased on the vascular endothelium of inflamed tissues, where it partakes in the rolling, firm adhesion and transmigration phases of leukocyte transmigration [5], [8], [9]. Interestingly, in addition to being an adhesin, VAP-1 is also an ecto-enzyme which belongs to the primary amine oxidases (also known as semicarbazide sensitive amine oxidases, SSAO; EC 1.4.3.21) [10]. Primary amine oxidases catalyze the conversion of primary amines to the corresponding aldehydes in a reaction, which concomitantly produces the highly potent bioactive substances hydrogen peroxide and ammonia. This enzymatic activity has been shown to be pivotal for the role of VAP-1 in the transmigration cascade [11]. The SSAO activity of VAP-1 has also been implicated in playing a role in various disease processes, like atherosclerosis and vascular complications in diabetes [12], [13]. In addition, it has been shown to be involved in glucose transport [14], adipocyte differentiation [15], and structural organization of vascular smooth muscle [16].

Alternative splicing (AS) of pre-mRNA plays a major role in post-transcriptional gene regulation by producing structurally and functionally distinct protein variants from the same genetic information. It has been estimated that 92–94% of all human multi-exon genes are in fact alternatively spliced [17]. The importance of AS has been well established in normal development and differentiation, during which a tightly controlled expression of specific protein isoforms is typical for many genes [17]–[20]. In addition to having qualitative effects on the cellular proteome AS plays a quantitative role in regulating the mRNA-levels of many genes. For example, alternative transcripts carrying a premature termination codon (PTC) are often directed for destruction using the nonsense-mediated degradation (NMD) pathway and, hence, less mRNA is available for translation. If, however, the aberrant transcript is translated, down-regulation of the corresponding protein levels may still occur via dominant-negative mechanisms [21]–[24].

We have cloned a shorter splice variant of VAP-1, VAP-1Δ3, from a human lung cDNA library, and used transient transfections, co-immunoprecipitations, and enzyme assays to elucidate the functional role of VAP-1Δ3. Here, we show that this isoform is widely expressed at the mRNA level and that the corresponding polypeptide does not display SSAO activity *in vitro*. Furthermore, we show that VAP-1Δ3 is able to dimerize with VAP-1 and suggest that the observed down-regulation of cell-surface expression and the diminished enzymatic activity of the full-length counterpart are caused by a dominant-negative effect due to heterodimerisation with the truncated isoform.

## Methods

### Ethics Statement

The use of the fetal tissues was approved by the Finnish National Authority for Medicolegal Affairs and the Regional Institutional Review Board of Medicolegal Affairs (Turun yliopiston eettinen toimikunta, Turku, Finland). Oral informed consent was obtained for the collection of samples and subsequent analysis. The use and documentation of oral consent was approved by the Institutional Review Board.

### Cells and Reagents

Human embryonic kidney fibroblast 239 cells (HEK293) and HEK293 cells constitutively expressing the Epstein-Barr Virus Nuclear Antigen (HEK293-EBNA) were cultured in Dulbecco’s modified Eagle’s medium (DMEM) (Gibco) supplemented with 10% fetal calf serum (FCS), 2 mM *L*-glutamine, sodium pyruvate, penicillin, and streptomycin.

Human umbilical vein endothelial cells (HUVEC) were isolated from human umbilical cord veins as described before [25] and cultured in RPMI 1640 medium containing 10% human AB-serum (Finnish Red Cross, Helsinki, Finland), 50 µg/ml endothelial cell growth factor (ECGF; Boehringer Mannheim GmbH), 5 U/ml heparin, 4 mM *L*-glutamine, penicillin, and streptomycin. Chinese hamster ovary (CHO) cells were grown in modified Eagle’s medium (MEM) with 10% FCS, 2 mM *L*-glutamine, penicillin, and streptomycin. Methylamine, benzylamine, p-tyramine, tryptamine, β-phenylethylamine, histamine, hydroxylamine and semicarbazide were from Sigma.

### Antibodies

The primary antibodies used for detecting the full-length VAP-1 and VAP-1Δ3 are listed in Table S1. In addition, the anti-VAP-1 antibody JG 2.10 was also used as biotinylated. Anti-flag mAb M2 (Sigma) and anti-myc mAb 9E10 (CRL1729 from ATCC) were used to detect the flag- and myc-tagged constructs, respectively. PAL-E (Abcam), anti-CD31 (M-20, Santa Cruz Biotechnology), anti-CD44 (Hermes-3/60D9, [26] and anti-CLEVER-1 (Alexa Fluor 488-conjugated 3–372, [27] antibodies were used to detect the vessels and the CD44 positive cell types in the tissue samples. Normal rabbit serum, normal goat serum, 3G6 (mouse anti-avian T cell antibody; [3]), AK1 (InVivo Biotech), mIgG2a (R&D Systems), and MEL-14 (rat anti-mouse L-selectin antibody, from E. Butcher) were used as negative controls. The secondary antibodies FITC-conjugated anti-goat Ig, anti-rabbit Ig, anti-mouse Ig, and anti-rat Ig were all from Sigma, Alexa Fluor 546-conjugated or Alexa Fluor 488-conjugated anti-mouse Ig and anti-rat Ig were from Invitrogen, and the PE-conjugated anti-mouse Ig and anti-rat Ig were from Southern Biotechnology. The Alexa Fluor 546 conjugated streptavidin was from Invitrogen.

### cDNA Cloning and Bioinformatics

A PCR clone of 771 bp covering nucleotides 38 to 808 of the VAP-1 cDNA (AF067406) was used as a probe to screen a human lung cDNA library in Lambda EMBL3 SP6/T7 vector (BD Biosciences Clontech). The positive plaques were screened with PCR using VAP-1 specific primers from exon 1 (5′-ACTCAGATCTCTACTCGCACT-3′) and exon 4 (5′-ATATGCAGAAAACCAGCTGTC-3′) to discard the plaques containing the previously known VAP-1 transcript. The amplification was carried out in Perkin-Elmer GeneAmp PCR System 2400 and the protocol was: step 1, 94°C for 5 min, step 2, 94°C for 1 min, step 3, 50°C for 1 min, step 4, 72°C for 1 min and step 5, 72°C for 5 min. Steps 2 to 4 were repeated for 29 times. The clones were sequenced on both strands by the sequencing service facilities of the Department of Medical Genetics, University of Turku (Turku, Finland). The nucleotide sequence obtained was submitted to GenBank (accession number JX020506).

Standard molecular biology techniques were used in plaque hybridization, lambda phage purification, *Escherichia coli* DH5α transformation and plasmid and phage DNA purifications. VAP-1Δ3 was further subcloned into pcDNA3.1 (Invitrogen), pIRES2-EGFP (Clontech), and pAdCMV [28] expression vectors for transfections. VAP-1Δ3 adenoviruses and control lacZ adenoviruses were produced as described earlier for VAP-1 [29], [30] at A.I. Virtanen Institute, Department of Medicine, University of Kuopio, Finland.

Sequence analyses and alignments were performed using the Wisconsin Package version 10.1-UNIX of the Genetics Computer Group (GCG; Madison, WI), SeqWeb of BioBox and EMBOS at Scientific Computing Ltd. (CSC; Espoo, Finland) and BLAST at NCBI (http://www.ncbi.nlm.nih.gov/BLAST/).

### RNA Expression

Multiple Tissue cDNA (MTC™) panels (BD Biosciences Clontech) of adult human tissues were used as templates in PCR. The PCR-primers used were located in exons 1 and 4 of the VAP-1 gene (*AOC3)*: The sequence for the sense primer was 5′-AAGCTGCTGGAGATGGAGGAGCAGG-3′ and for the antisense primer 5′-CCTCGGAAGTAGATGGAGTCGGCAGAGT-3′. GAPDH primers provided by the supplier were used for normalization. The amplification was carried out in a Perkin-Elmer GeneAmp PCR Systems 2400, as suggested by the cDNA panel supplier. The protocol was: step 1, 94°C for 1 min, step 2, 94°C for 30 s, step 3–4, 68°C for 2 min and step 5, 72°C for 5 min. Steps 2 to 4 were repeated for 34 times.

Total RNA was prepared from human fetal skeletal muscle, liver, lung, brain, heart, kidney, skin, cartilage and tonsil around age 20 weeks of gestation by using the guanidinium isothiocyanate method [31]. Reverse transcription and PCR were performed in a one step reaction by using GeneAmp® Gold RNA PCR Reagent Kit (PE Applied Biosystems). The thermal cycles of amplification were carried out in a Perkin-Elmer GeneAmp PCR Systems 2400 and the protocol was according to the manufacturer’s instructions for one step reverse transcriptase-PCR (RT-PCR). One microgram of total RNA and 20 pmol/µl of primers were used in all reactions. The sequences for the specific sense and antisense primers are the same that were used with the MTC-panels (see above). β-actin primers used for normalization were from Stratagene. The Southern blot analysis was performed of the RT-PCR reactions, which were separated on agarose gels and blotted onto Hybond N filters. The probe recognizing the amplified sequence was generated from the original VAP-1 cDNA by PCR using the same primers as in the RT-PCR reactions. Standard methods in molecular biology were used in Southern blotting, hybridization and autoradiography.

The Multiple Tissue cDNA panels of adult human tissues were also used as templates in quantitative PCR (qPCR). Universal ProbeLibrary Assay Design Center (Roche Applied Science; https://www.roche-applied-science.com/sis/rtpcr/upl/adc.jsp) was used to design probes and primers that would distinguish between the two VAP-1 splice variants. Universal ProbeLibrary #12 targets exon 3 of the AOC3 gene that is missing from VAP-1Δ3 (detects the full-length mRNA) and Probe #55 anneals to the alternatively formed exon2/exon4 junction (detects VAP-1Δ3 mRNA). TaqMan® Gene Expression Assay for human β-actin (endogenous control) was from Applied Biosystems. The PCR reactions were prepared as suggested by the supplier and run on Applied Biosystems 7900HT Fast Sequence Detection System in the Finnish DNA Microarray Center, Centre for Biotechnology, Turku, Finland. The quantitative PCR runs were performed with triplicates of each sample. Changes in cycle threshold levels (ΔC_T_) were calculated by subtracting the average of β-actin C_T_ values from the average of target gene C_T_ values. Relative expression of the gene analyzed was estimated as in [32] using the formula: relative expression  = 2^−ΔCT^, where ΔC_T_ = C_T_ (target gene) − C_T_ (β-actin). The quantity of VAP-1 and VAP-1Δ3 mRNAs was expressed as percentage of β-actin mRNA after multiplying relative target gene expression by a factor of 100.

### In vitro Translation

In vitro translation was performed using TNT® Quick Coupled Transcription/Translation System from Promega. The cDNA clones for the full-length VAP-1 and VAP-1Δ3 in pcDNA3.1 were used as templates. ^35^S labeled methionine (>1,000 Ci/mmol at 10mCi/ml; Amersham International) and 1 µg of template was used for each transcription/translation reaction. The protocol used was as suggested by the manufacturer. The translation reactions were precipitated using a polyclonal anti-VAP-1 antibody coupled to the Protein-A-Sepharose beads CL-4B (Amersham International). The beads were prepared and the procedure performed according to manufacturer’s instructions. The concentration for the anti-VAP-1- antibody was 20 µg/ml. 5 µl of each sample was loaded onto a SDS-PAGE gel and the dried gel was exposed on Kodak X-Omat film overnight.

### Transfections

HEK293 and HEK293-EBNA cells were transiently transfected using either the calcium phosphate method or Fugene HD (Roche Applied Science). For the calcium phosphate procedure 5–20 µg of full-length VAP-1 or VAP-1Δ3 in pcDNA3.1 was used for a 90 mm plate and the cultures were incubated for 24 to 48 hours. With Fugene transfection reagent, 2 µgs of DNA was used for a 6-well plate well with 3–8 µl of the reagent.

HUVECs were infected with the adenoviral VAP-1 and VAP-1Δ3 constructs as described before [29]. Briefly, confluent HUVECs were incubated with 400–1200 plaque forming units (pfu) of virus for ½-2 hours. The medium was removed, the plates washed and the cells were stained or lysed the next day with 0.2% Nonidet P-40 (Fluka Chemie AG) in PBS for 2 h at +4°C.

Stable VAP-1 expressing CHO cells were transfected either with the VAP-1Δ3 in pIRES2-EGFP or the pIRES2-EGFP vector (control) using Fugene HD as above.

### Cell Sorting

Stable VAP-1 expressing CHO cells were transfected either with the pIRES2-EGFP vector without an insert or with pIRES2-EGFP-VAP-1Δ3. The cells were cultured and sorted based on the VAP-1 and the enhanced green fluorescent protein (EGFP) expression with FACSVantage SE with DiVa options cell sorter (Becton Dickinson) in the Cell Imaging Core at the Centre for Biotechnology, Turku, Finland.

### Immunostainings

For flow cytometry the transfected cells were detached with trypsin and washed with PBS. For intracellular staining the cells were permeabilized with acetone. Cell suspensions of acetone-permeabilized and non-permeabilized cells were stained using the polyclonal anti-VAP -1 or the monoclonal JG2.10 antibody, and the appropriate secondary FITC conjugated antibody. Normal rabbit serum and MEL-14 were used as negative controls. The stained cells were analyzed using FACScan or FACSCalibur and the CellQuestPro, Win MDI or Cyflogic softwares.

Cover slips in a 24-well plate were plated with HEK293 cells and transfected either with 0.25 µg VAP-1-pcDNA3.1 or with the same amount of VAP-1Δ3-pcDNA3.1. Aliquots of the cells were permeabilized with acetone for intracellular staining. All cells were stained with the polyclonal anti-VAP antibody and the appropriate secondary FITC conjugated antibody. Finally, the cover slips were mounted with Fluoromount-G (Southern Biotechnology Associates) and analyzed with fluorescence microscopy.

Frozen tissue sections of lymph node, tonsils, liver, skin, appendix, retina, kidney, and gut were acetone fixed and stained with the anti-VAP-1 antibodies 2D10 and JG 2.10, anti-CD44, anti-CD31, anti-CLEVER-1 and PAL-E, or with the negative control antibodies MEL-14, AK-1, mIgG2a and normal goat serum followed by PE-, FITC-, Alexa Fluor 546- or Alexa Fluor 488-conjugated secondary antibodies. With the biotinylated JG 2.10 Alexa Fluor 546 conjugated streptavidin was used as a secondary reagent. Fluoromount-G or ProLong Gold antifade agent (Life Technologies) was used as a mounting medium and the analysis was performed with fluorescence microscopy.

### Co-immunoprecipitations

HEK293 and HUVEC cells were co-transfected with the full-lengthVAP-1 subcloned into pRK5flag [33], together with either a myc-tagged version of VAP-1Δ3 subcloned into pTB399 [34], or with the corresponding empty vectors (control). After an overnight incubation, the cells were lysed in 700 µl of ice cold lysis buffer of 50 mM Tris-HCl, 150 mM NaCl, 1% Nonidet P-40 (Fluka Chemie AG), complete EDTA-free protease inhibitor cocktail tablet (Roche), 1 mM Na_3_VO_4_, 10 mM NaF, 1 mM β-glyserophosphate, 5 mM CaCl_2_ and 5 mM MgCl_2_. The lysate was pre-cleared by shaking for 20 min with Protein G Protein G Sepharose™ 4 Fast Flow beads (GE Healthcare). Next, two aliquots of the supernatant were incubated with Protein G beads together with either the anti-flag mAb M2 or the anti-myc mAb 9E10 for 1 h at +4°C. After 3 to 5 washes with the lysis buffer, Laemmli’s sample buffer with 5% 2-mercaptoethanol was added to the beads. Thereafter the samples were separated in 6–12% SDS-PAGE gels and immunoblotted with Amersham™ Enhanced Chemiluminescence method (ECL) (GE Healthcare) using the anti-myc and anti-flag ab’s.

### Enzyme Assays

The transfected cells were trypsinized and lysed in 0.2% Nonidet P-40 in PBS for 2 h at +4°C with rocking. The supernatant was collected after centrifugation (30 min at +4°C, 12 000 g). The transfection efficiencies were assayed by immunofluorescent staining (see above) and the protein concentration was measured with Bio-Rad *DC* Protein Assay (Bio-Rad Laboratories). The SSAO activity of the cell lysate was measured via detecting the H_2_O_2_-production with the Amplex Red reagent (10-acetyl-3,7- dihydroxyphenoxazine; Molecular Probes Europe BV). Cell lysates were preincubated in Krebs-Ringer Phosphate Glucose (KRPG) for 30 min at 37°C with or without the specific SSAO inhibitor semicarbazide (100–1000 µM). Catalytic reactions were initiated by adding various substrates, all at 1 mM, and the H_2_O_2_-detecting mixture containing horseradish peroxidase and Amplex Red reagent. Fluorescence intensity of the samples was measured with Tecan ULTRA or Tecan Infinite microplate reader (excitation, 545 nm; emission, 590 nm).

The H_2_O_2_ concentration was calculated from calibration curves based on serial dilutions of standard H_2_O_2_. To evaluate the amount of H_2_O_2_ formed in the SSAO mediated reaction, H_2_O_2_-production in control wells (with the SSAO inhibitor) was subtracted from the total amount of H_2_O_2_ formed. All the assays were performed as duplicates.

### Statistical Analysis

Statistics were performed using Student’s *t* test. Values of P<0.05 were regarded as statistically significant.

## Results

### A Shorter Transcript of VAP-1 is Expressed in Several Human Tissues

**Figure 1:**
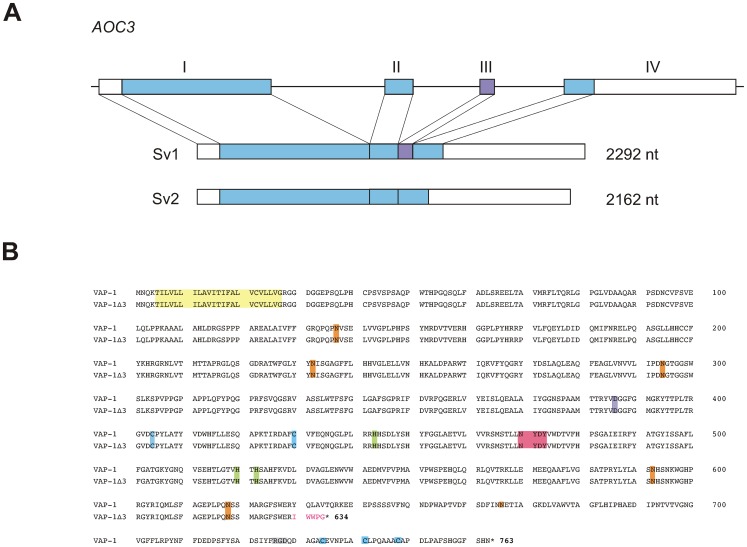
Characteristics of the alternatively spliced transcript of VAP-1. (**A**) Schematic presentation of the exon–intron organization of the human VAP-1 gene, *AOC3*. The boxes with roman numerals (I-IV) represent the exons. The translated regions are shown in *color* and the 5′- and 3′-untranslated regions in *white*. Exon III (*violet*) is spliced out in the shorter splice variant. Sv1: the full-length splice variant; Sv2: the alternatively spliced shorter splice variant. (**B**) Sequence alignment of the two VAP-1 isoforms. The deduced amino acid sequences of VAP-1 and the shorter isoform VAP-1Δ3. Highlighted are: *light yellow,* the hydrophobic N-terminal sequence; *pink*, the conserved signature motif of the active site, in which the first tyrosine is post-translationally modified to topaquinone; *lilac*, the (putative) catalytic site base; *light green*, the conserved Cu(II) binding histidine residues; *light blue*, the conserved cysteine residues involved in dimerization; *orange* the putative N-linked glycosylation sites, *grey*, RGD sequence. The amino acids unique to VAP-1Δ3 are in red.

In order to search for additional VAP-1-related transcripts in human tissues, a lung cDNA library was screened with a VAP-1-specific probe. Four of the clones thus found were identical to the closely related primary amine oxidase AOC2 [33] and two represented a 130-bp shorter splice variant of VAP-1. By comparing the nucleotide sequence of this shorter transcript with that of *AOC3,* the gene for human VAP-1, it could be deduced that the transcript lacked the sequence corresponding to the third exon of *AOC3* suggesting that the shortening was due to alternative splicing of the primary transcript. For this reason the shorter splice variant was designated VAP-1Δ3. (Figure 1A, Figure S1). The corresponding polypeptide chain would, in turn, be shortened by 129 amino acids due to the split codon at the exon2-exon3 interface and the emergence of a premature termination codon in exon4 resulting from skipping exon3. This C-terminal truncation results in the deletion of an Arg-Gly-Asp (RGD) - tripeptide, three of the five cysteine-residues, and two of the putative glycosylation sites that are found in the full-length VAP-1 protein. In the active site proper, however, most of the conserved motives essential for the SSAO activity of VAP-1 remain intact. (Figure 1B).

**Figure 2:**
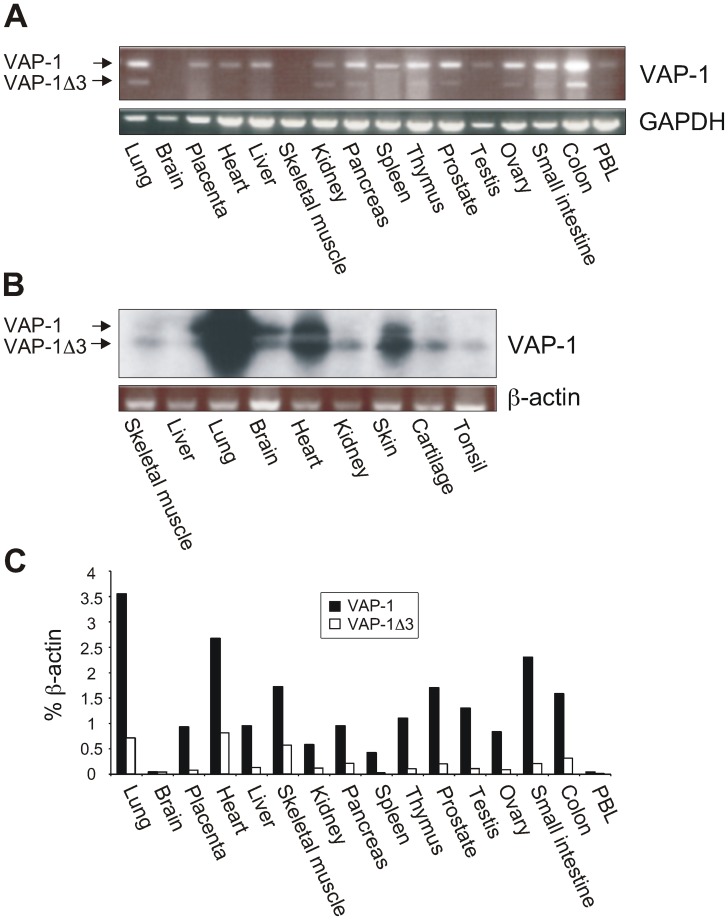
Expression of VAP-1Δ3 mRNA in adult and fetal human tissues. (**A**) Commercial first strand cDNA panels were used to determine the mRNA expression of VAP-1Δ3 in respect to the expression of the full-length VAP-1 in adult human tissues by PCR. GAPDH expression was used as an endogenous control. (**B**) RT-PCR analysis of nine fetal human tissues was performed using VAP-1 and β-actin specific primers. The identity of the resulting amplicons was verified with Southern blotting using a VAP-1-specific probe. The two alternatively spliced mRNA species are marked with *arrows*. (**C**) qPCR analysis was performed with transcript specific primers and probes using the commercial first strand cDNA panels as templates. The expression levels of VAP-1 and VAP-1Δ3 are presented as percentages of β-actin mRNA expression in the same sample.

The expression pattern of VAP-1Δ3 mRNA was characterized by RT-PCR using commercial first strand cDNA panels of adult human tissues and human fetal RNAs as templates. The results showed that VAP-1Δ3 mRNA was expressed in most of the human tissues studied in which the full-length VAP-1-transcript was detected. However, in some tissues (placenta, heart, liver, spleen, and testis) the shorter transcript was not detectable with RT-PCR (Figure 2A). Because the corresponding mRNA-levels in the fetal tissues were even lower than those in the adult samples, the gels with the RT-PCR amplicons were further Southern blotted and hybridized with a VAP-1-specific probe in order to amplify the signal and verify the specificity of the PCR-bands. Surprisingly, in fetal tissues VAP-1Δ3 mRNA seems to be the predominant transcript in kidneys, cartilage and tonsils. Also of interest is that in contrast to the lack of VAP-1 expression in the brain sample of the adult tissue cDNA-panel, both VAP-1-splice variants are detectable in the fetal brain RNA. (Figure 2B). To quantify the relative expression levels of VAP-1Δ3 mRNA in the adult tissues we performed qPCR using splice form specific primers and probes (Figure 2C). In contrast to the results obtained with conventional RT-PCR the more sensitive qPCR revealed the expression of VAP-1 mRNA in skeletal muscle. Furthermore, small amounts of VAP-1Δ3 mRNA were detected with qPCR also in those tissues that originally seemed to lack the shorter transcript. The highest levels of VAP-1Δ3 in respect to the full-length transcript were detected in skeletal muscle, heart, pancreas, kidney, and lung (33–20% of full-length mRNA expression).

### The Polypeptide Translated from VAP-1Δ3-cDNA is Detectable with VAP-1-Antibodies

In order to find out if the cDNA-clone for VAP-1Δ3 is able to produce a stable polypeptide chain identifiable with VAP-1 antibodies, coupled *in vitro* transcription/translation was performed. Translation of the mRNA transcribed from the VAP-1Δ3-cDNA did result in the production of a polypeptide chain of the expected size (634 amino acids) that could be detected using a polyclonal antibody for VAP-1 (Figure S2A). This antibody was subsequently used to detect the VAP-1Δ3-polypeptide in the transfection experiments. In addition, several monoclonal anti-VAP-1 antibodies were tested for their ability to detect VAP-1Δ3 in order to find an antibody that could discriminate between the two isoforms. Most of these monoclonals proved to be able to recognize both isoforms, the only exception being JG 2.10 that was specific for the full-length VAP-1 molecule (Table S1 and Figure S2B) suggesting, thus, that the epitope recognized by JG 2.10 resides within the carboxyterminal part of the molecule that is missing from VAP-1Δ3.

### The Subcellular Localization of Transfected VAP-1Δ3 Depends on the Host Cell

**Figure 3:**
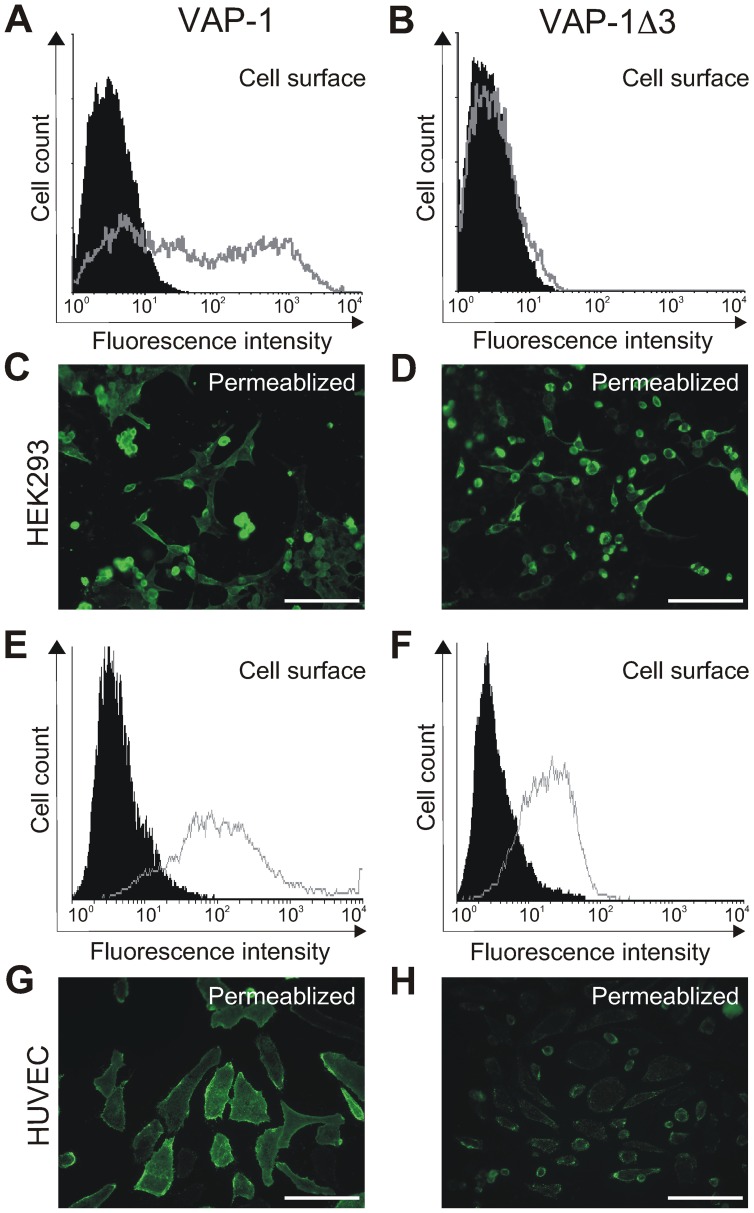
Expression of VAP-1Δ3 in transiently transfected cell lines. Flow cytometry of HEK293 cells transfected either with the full-length VAP-1- or with VAP-1Δ3 -cDNAs in pcDNA3.1 (**A–B**). The *gray* histograms: staining with the anti-VAP-1 polyclonal antibody; the *black* histograms: staining with a negative control antibody. The expression was also examined by fluorescence microscopy of acetone-permeabilized coverslip-plated HEK293 cells transfected with the corresponding constructs (**C–D**). In **E–F**, flow cytometry of HUVECs infected with pAdCMV-constructs of VAP-1- and VAP-1Δ3. The *gray* histograms: staining with the anti-VAP-1 polyclonal antibody; the *black* histograms: staining with a negative control antibody. The expression was also examined by fluorescence microscopy of acetone-permeabilized coverslip-plated HUVECs infected with the corresponding constructs (**G–H**). Scale bar 100 µm.

The VAP-1Δ3-cDNA was further subcloned into the expression vectors pcDNA3.1 and pAdCMV in order to study the characteristics of the corresponding protein *in vitro*. FACS analysis with VAP-1-detecting antibodies showed that when transiently transfected into HEK293 cells, VAP-1Δ3 was not detected on the surface of the host cells, unlike the full-length VAP-1 in the correspondingly transfected cells (Figure 3A-B). To rule out the possibility that VAP-1Δ3 was not translated from the transfected cDNA in HEK293 cells, the transfectants were also plated on coverslips for fluorescence microscopy. After permeabilizing the cells with acetone the presence of VAP-1Δ3 was clearly visible in the cytoplasm of the transfected cells (Figure 3C–D). In contrast, both VAP-1 and VAP-1Δ3 were detected on the cell surface as well as in the cytoplasm of HUVECs, which had been infected with the adenoviral constructs (Figure 3E–H), even though protein expression is driven by a cytomegalovirus (CMV) promoter in both vector-types and the constructs carry identical insert sequences. Neither of the cell types used expresses VAP-1 endogenously. However, being endothelial cells, HUVEC may be a more natural host for a cell-surface-destined adhesion molecule and equipped with a different sort of machinery for protein targeting, which would explain the differences observed between the cell-surface-expression of VAP-1Δ3 in the two cell lines. The results thus demonstrate that the protein produced from VAP-1Δ3-cDNA is stable enough to be detected inside the cells and that the cell line used as a transfection host determines whether or not the isoform is targeted to the cell surface.

### The Shorter Isoform of VAP-1 cannot be Detected without the Full-length Protein in the Tissues Studied

Due to the fact that only the last five C-terminal amino acids of the truncated polypeptide chain are unique to VAP-1Δ3 (Figure 1B), it proved to be impossible to produce an antibody that would recognize only the shorter isoform of VAP-1. For this reason, in order to be able to study the expression pattern of VAP-1Δ3, we used different combinations of earlier produced monoclonal antibodies for VAP-1, most of which were able to detect VAP-1Δ3 as well (see above). Frozen sections of several human tissues (lymph node, inflamed and non-inflamed tonsils, liver, skin, appendix, retina, kidney, and gut) were stained with the monoclonal antibody JG 2.10, which recognizes only the full-length VAP-1, and with 2D10, which is able to detect both isoforms (Table S1). The staining patterns with the two antibodies in all tissues were identical and typical for VAP-1 indicating that VAP-1Δ3 was not present independently of full-length VAP-1 in any of the tissues studied (examples of the staining pattern in human tissues are shown in Figure S3, S4, S5).

### VAP-1Δ3 does not Display SSAO Activity

**Figure 4:**
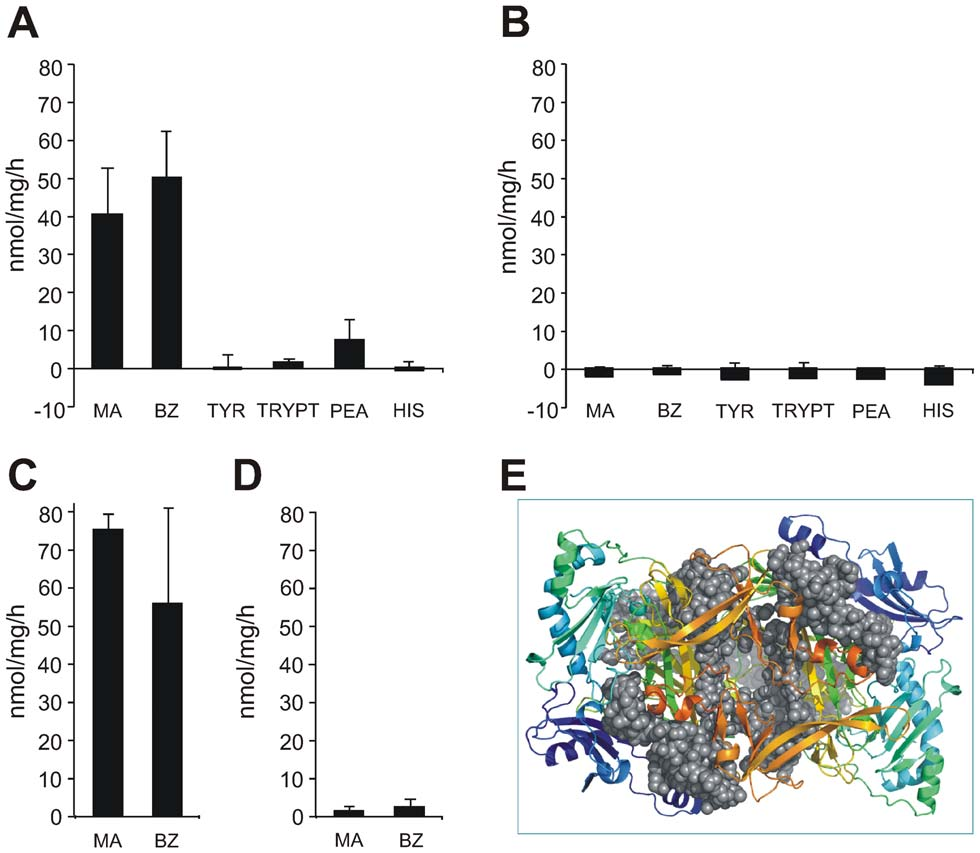
Enzymatic activities of the two VAP-1 isoforms. The SSAO-activity and substrate specificities of VAP-1 and VAP-1Δ3 were defined by a fluorometric assay from lysed transfectants. (**A**) HEK293 cells transfected with VAP-1. (**B**) HEK293 cells transfected with VAP-1Δ3 (**C**) HUVECs infected with VAP-1 (**D**) HUVECs infected with VAP-1Δ3. The final concentrations of all substrates were 1 mM. *MA* methylamine; *BZ* benzylamine; *TYR p-*tyramine; *TRYPT* tryptamine; *PEA* 2-phenylethylamine; *HIS* histamine. The results are expressed as nmol/mg/h+standard deviation (SD) (n = 3). (**E**) The structure of dimeric VAP-1 (PDB code 1US1; Airenne et al., 2002), viewed from the side of the carboxy-terminus along the two-fold axis. Both monomers are drawn in rainbow colors from blue amino-termini to red carboxy-termini. The amino acids missing from VAP-1Δ3 (aa 634-761) are illustrated as gray spheres of different shades. The picture was generated with PyMol [54].

VAP-1 has been shown to exhibit SSAO activity, which contributes to its role in leukocyte trafficking [11]. Since most of the conserved active site amino acid residues essential for SSAO activity are still intact in VAP-1Δ3, we wanted to study, whether VAP-1Δ3 displays enzymatic activity as well. Several substrates were used in Amplex Red assays to look for SSAO activity in lysates of HEK293 cells transfected with VAP-1Δ3. Neither when using methylamine as a substrate nor when using benzylamine, the two best substrates of VAP-1, could any SSAO-activity be detected in the VAP-1Δ3-transfectants. Since a closely related SSAO, AOC2, prefers larger amine substrates and has negligible activity towards the smaller amines [33], we also used tyramine and tryptamine as substrates. No SSAO activity was, however, detected in these experiments either (Figure 4 A–B). To rule out a cell type -specific inhibitory effect on the putative enzymatic activity, we also tested HUVECs infected with the adenoviral VAP-1Δ3-construct for SSAO activity. Even though the corresponding VAP-1-expressing HUVECs were enzymatically active, the VAP-1Δ3 expressing ones were again devoid of SSAO activity (Figure 4C–D).

To get an insight into the underlying cause for the enzymatic inactivity of VAP-1Δ3, we compared it to the published structures of human VAP-1 [35]–[37]. (Figure 4E) The carboxy-terminally truncated VAP-1Δ3 lacks the last 129 amino acids of the D4 domain of the mushroom shaped VAP-1. Two stretches of these missing residues lie on the protein surface: the first (aa 634–674) is close to the D2 domain, and the second (aa 723–763) forms the platform of the heart shaped dimer. The rest of the missing residues, however, form two long beta sheets at the monomer-monomer interface. Moreover, the missing part of the protein includes the strictly conserved His684, which takes part in coordinating the active site copper essential for SSAO activity [37], [38]. Thus, even though the active site residues *per se* are still intact, the enzymatic activity of VAP-1Δ3 is most likely affected due to the lack of His684 and the effect the missing amino acid stretches have on the formation of a proper conformation in the active site cavity.

### VAP-1Δ3 Down-regulates the Cell-surface Expression and Enzymatic Activity of VAP-1

**Figure 5:**
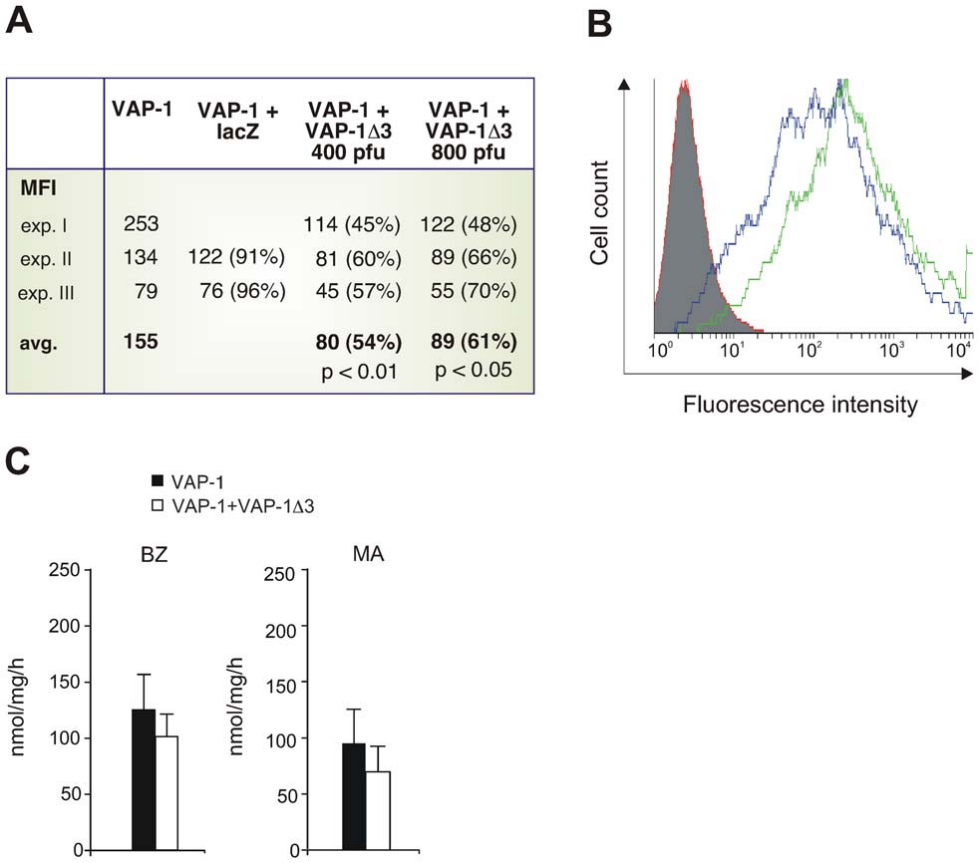
VAP-1 cell-surface expression and SSAO activity of transfectants expressing both VAP-1 and VAP-1 Δ3. HUVECs were first infected with adenoviruses carrying the cDNA for the full-length VAP-1 and then with those carrying the VAP-1Δ3-cDNA. LacZ adenoviruses were used as co-transfection controls. VAP-1 expression was determined by FACS using the antibody JG 2.10. (**A**) The surface expression of VAP-1 in the transfected cells as mean fluorescence intensities (MFIs) and the averages of MFIs from three experiments. In parentheses, the percentage of VAP-1 surface expression in the co-transfected cells compared to the cells transfected only with VAP-1 ( = 100%). VAP-1Δ3 adenoinfection was performed with two different doses, 400 and 800 pfu (**B**) A histogram of a representative experiment. The number of cells is shown in the y-axis and the fluorescence in the x-axis. The *green* histogram shows VAP-1 surface expression in the cells co-infected with the control lacZ adenovirus, the *blue* histogram shows VAP-1 expression in the cells co-infected with VAP-1Δ3, and the *red* histogram shows the staining with a negative control antibody. (**C**) SSAO activity of stably transfected VAP-1-CHO cells co-transfected either with the EGFP-IRES2 empty vector (black) or with the same vector carrying VAP-1Δ3 (white). The enzymatic activity of lysed transfectants was determined in fluorometric assays. The substrates used were *BZ* benzylamine; *MA* methylamine (1 mM). Results are shown as nmol of H_2_O_2_/mg/h+SEM. The experiment was repeated four times with MA and five times with BZ.

To study the consequences of simultaneous expression of the two VAP-1-isoforms in the same cell, HUVECs were infected first with adenoviral constructs of VAP-1 and subsequently with those carrying VAP-1Δ3. The surface expression of full-length VAP-1 was deduced from the FACS analysis of co-infected cells stained with VAP-1-recognizing antibodies. When compared to transfectants containing only the full-length molecule, the presence of VAP-1Δ3 significantly affected the cell surface expression of VAP-1: the average reduction was 39% with 800 pfu of adenoviral construct (p<0.05) and 46% with 400 pfu (p<0.01) (Figure 5A–B). As a control, the VAP-1 expressing cells were co-infected with adenoviral constructs carrying lacZ instead of VAP-1Δ3. In this case, co-infection did not result in a similar reduction of the surface expression of VAP-1.

Next, CHO-cells transfected with VAP-1 and stably expressing the protein, were co-transfected with VAP-1Δ3 in EGFP-IRES2-vector or with the empty EGFP-IRES2-vector (control). The transfected cells were sorted three times based on the EGFP-expression to be able to expand the cell population expressing both VAP-isoforms. Finally, SSAO activity was determined from lysates of co-transfected cells expressing both VAP-1 and EGFP using methylamine and benzylamine as substrates. The data showed a trend towards diminished SSAO activity in the lysates of VAP-1Δ3-co-transfected cells when compared to the control transfected cells, even though the results did not reach statistical significance (Figure 5C).

### VAP-1Δ3 is Able to form Heterodimers with the Full-length Protein *in vitro*

**Figure 6:**
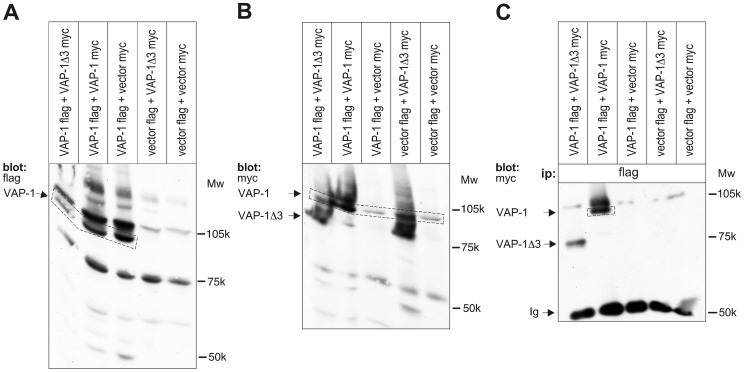
Heterodimerisation of VAP-1 and VAP-1 Δ3. Lysates of HEK293 cells co-transfected with flag-tagged VAP-1 cDNA, myc-tagged VAP-1Δ3 cDNA or with the corresponding tagged empty vectors in different combinations were separated in SDS-PAGE (with or without prior immunoprecipitation) and blotted to nitrocellulose membranes. The functionality of the tagged constructs and the ability to detect the proteins with corresponding antibodies was first verified by using the flag-antibody (A) or the myc-antibody (B) in control gels without prior immunoprecipitations. Aliquots of the same lysates were then immunoprecipitated with the flag antibody prior to gel electrophoresis, and the immunoprecipitated product was detected using the myc-antibody (**C**). The sizes of the two VAP-1 isoforms and the molecular weight markers are indicated. *Ip* Immunoprecipitation, *Ig* Immunoglobulin.

VAP-1 is present on the cell surface as a homodimer [4], [39]. In cells that express both the full-length molecule and the shorter splice variant, heterodimerization of the two different-sized monomers may occur and result in changes in the cell surface expression and/or function of VAP-1. The observed down-regulation of VAP-1 surface expression in the VAP-1-VAP-1Δ3-co-transfectants prompted us to further explore this possibility and perform co-immunoprecipitations in lysed HEK293 cells co-transfected with flag- and myc-tagged VAP-1 and myc-tagged VAP-1Δ3 cDNAs (Figure 6). When immunoprecipitation was performed with a flag-antibody, the myc-tagged VAP-1Δ3 was seen to co-immunoprecipitate with the flag-tagged full-length VAP-1. Control co-precipitations with the corresponding vectors without an insert confirmed the specificity of the observed interaction. Due to the lack of a specific antibody recognizing only the shorter VAP-1Δ3-polypeptide, we could not study the heterodimerization with the corresponding endogenous proteins.

## Discussion

In resting conditions VAP-1 is stored in intracellular vesicles from where it is rapidly translocated to the luminal surface of endothelial cells upon inflammation [5]. When on the cell-surface, VAP-1 takes part in the leukocyte transmigration cascade using both its adhesive and enzymatic properties [4], [11]. The regulation of the induced cell surface expression and the ultimate shutting down of the response are, however, less well characterized.

Alternative splicing of pre-mRNA results in the production of various protein isoforms from the same genetic information allowing of different functional and regulatory properties for the individual forms. Here, we have shown that alternative splicing of VAP-1 pre-mRNA brings about two mRNA-species differing in size yet producing polypeptide chains that are able to heterodimerize. Since VAP-1 is known to function as a dimer, heterotypic pairing would result in a dominant negative effect by interfering with the formation of prototypic VAP-1-homodimers. Furthermore, VAP-1- VAP-1Δ3-dimers seem to be retained inside the cell and excluded from proper targeting to the cell membrane, since the amount of VAP-1 detectable on the cell surface with anti-VAP-1 antibodies is reduced in cells co-transfected with VAP-1Δ3 and VAP-1 cDNAs. Alternatively, the heterodimers are on the cell surface but the dimerization masks functional epitope(s) of VAP-1 and renders them undetectable with the used antibodies. The production of the shorter VAP-1Δ3-isoform could thus serve as a regulatory mechanism whereby leukocyte trafficking can be tuned down via reducing the number of functional cell-surface VAP-1 molecules available for facilitating transmigration.

It is highly probable that heterodimerization with VAP-1Δ3 affects the enzymatic activity of VAP-1 as well. Omission of a considerable part of the full-length VAP-1-polypeptide chain in VAP-1Δ3 makes it very unlikely that a heterodimer between these two isoforms would adopt a similar conformation to that of a VAP-1 homodimer. Furthermore, the formation of a functional catalytic site in a heterodimer is extremely unlikely, as one of the copper coordinating residues and several amino acids partaking to the formation of the enzymatic cavity are missing. Indeed, the data obtained from measurements of SSAO-activity in lysates of VAP-1-VAP-1Δ3-co-transfected cells show that there is a trend towards reduced enzyme activity when compared to the control VAP-1-transfected cells. Since SSAO-activity is an integral part of the functional modality of VAP-1, the effect on leukocyte trafficking is thus potentiated by both arms of the functionalities of VAP-1 - adhesion and SSAO-activity - being compromised by heterodimerization with VAP-1Δ3. Since VAP-1 is indicated in playing a role also in other physiological and pathological processes, presumably through the SSAO-activity, the effect VAP-1Δ3 exerts on the functional properties of full-length VAP-1 might have consequences also on other disease entities, like atherosclerosis and diabetes [12], [13].

It is essential for a faithful reproduction of genetic information into correctly functioning polypeptides that cells can distinguish between aberrant mRNAs resulting from errors in transcription or splicing and those that are correctly transcribed. Nonsense-mediated mRNA decay (NMD) is a quality-control surveillance mechanism whereby mRNA molecules carrying a premature termination codon (PTC) can be recognized and degraded [40], [41]. The mammalian NMD-machinery is part of the pre-mRNA splicing apparatus and functions during the initial “pioneer” round of translation, where a set of proteins mark the exon-exon junctions after splicing. This exon-junction complex (EJC) is displaced by ribosomes during translation, and according to the prevailing view, is essential in determining, which mRNA molecules are targets for NMD. Why is it then that the premature termination codon in VAP-1Δ3 does not lead to degradation of the aberrant mRNA? The answer may lie in the observation that the ability of a PTC to bring about NMD seems to be position dependent: it is believed to be activated when the stop codon is situated >50–55 nt upstream of the 3′-most splicing-generated exon-exon junction [42]. When a stop codon is encountered, other RNA-binding proteins surveying translation bind to the mRNA template and check for the presence of possible downstream EJCs. If a downstream EJC is detected, the stop codon is classified as premature and the template mRNA destroyed via NMD. The PTC of VAP-1Δ3, however, is located in the terminal exon of VAP-1, 15 nt downstream of the last exon-exon junction, and therefore is not a classical target for the NMD pathway.

There are several examples of alternatively spliced transcripts carrying a PTC that are not destroyed but give rise to truncated proteins. Such proteins have been implicated in playing a role in post-transcriptional gene regulation, but also in causing disease, through acting as mainly dominant negative effectors [43]–[49]. Mechanistically, the adverse effects caused by dominant negative isoforms are the results of aberrant dimerization, incorrect subcellular targeting and altered ability either to bind ligands, to signal, or to catalyze enzymatic reactions [49], [50].

A splice variant that acts as a dominant negative modulator by dimerizing with the wild type isoform and down-regulating its expression has been reported, for example, for human EMR2, a myeloid cell –specific EGF-TM7 receptor [47]. On the other hand, human glucocorticoid receptor isoform β (GRβ) is an example of a transcript variant that results in a production of a C-terminally truncated protein, which functions as a dominant negative effector of the full-length receptor GRα via several mechanisms including heterodimerization. The increased production of this variant has been suggested to result in a tissue-specific insensitivity to glucocorticoids in disorders like rheumatoid arthritis and systemic lupus erythematosus [51]. Isoforms with truncated C-termini exist for many other nuclear receptors as well, most of which display dominant-negative activity on the transactivational properties of their full-length counterparts and all of which are unable to bind their endogenous ligands [52]. The recently cloned histamine H4 receptor variants are further examples of dominant negative modulators: hetero-oligomerization between the different isoforms results in intracellular retention of the receptor molecules [53].

The facts that VAP-1Δ3 mRNA is processed into polyA-containing mature transcripts abundantly enough to be found from a lung cDNA library and that the shorter splice variant can be found in several human tissues, further support the view that VAP-1Δ3 mRNA is indeed able to escape from the NMD machinery and play an important role in the post-transcriptional regulation of VAP-1 expression. Further studies are needed to test, whether the shorter splice variant of VAP-1 has a specific immunomodulatory role in terminating inflammatory processes by regulating the amount of bioactive VAP-1 on the cell surface and, thus, tuning down leukocyte extravasation. Furthermore, the predominance of VAP-1Δ3 in certain fetal tissues suggests that its regulatory role is underlined during fetal life and that it may have additional as of yet unknown functions in development and differentiation.

## Supporting Information

Figure S1 **Aligned nucleotide sequences of VAP-1 and VAP-1Δ3.** VAP1 represents the full length VAP-1 and VAP1Δ3 the alternatively spliced shorter transcript of VAP-1. The translation start codon (highlighted in yellow) and the stop codons (red) are indicated.
(DOC)

Figure S2 **In vitro transcription/translation and immunostainings of the two VAP-1 splice variants.** (**A**) *In vitro* transcription/translation reactions were performed using the two VAP-1 splice variant cDNAs or the empty vector (control) as templates. For additional size controls, the two AOC2 splice variant clones described before in [33] were also used as templates. The resulting polypeptides were immunoprecipitated with a polyclonal VAP-antibody and run in SDS-PAGE. The size of the monomeric VAP-1 chain (90 kDa) is indicated. (**B**) HEK293 cells transfected with either the full-length VAP-1 or with VAP-1Δ3 and stained with TK 8–14 and JG 2.10.
(TIF)

Figure S3 **Expression of VAP-1 isoforms in human tissues.** Frozen sections of gut (**A**), tonsil (**C**), and kidney (**E**) were stained with JG 2.10, which detects only the full-length isoform of VAP-1. (**B, D, F**) The corresponding tissues stained with 2D10 which detects both VAP-1 isoforms. In all, the typical VAP-1 staining pattern in the blood vessels can be seen. In B, intestinal villi, in D blood vessels, and in F a glomerulus, are pointed out by arrows.
(PDF)

Figure S4 **Expression of VAP-1 and various cell type markers in human tissues.** Frozen sections of peripheral lymph nodes (A and C) and tonsils (B and D) were stained with JG 2.10, which detects VAP-1, and with other antibodies recognizing particular cell type markers to verify the localization of VAP-1. The markers used are: CD31 and PAL-E for vascular endothelium, CD44 as a pan-cellular marker (excluding high endothelial venules), and CLEVER-1 for lymphatic endothelium. In A, the co-localization of CD31 and VAP-1, and in D, the co-localization of PAL-E and VAP-1 has been shown on blood vasculature. In C and D, VAP-1 is seen not to co-localize with CD44 or CLEVER-1. The inserts in the lower left hand corner depict staining with the negative control antibody. Scale bar 50 µm.
(PDF)

Figure S5 **Expression of VAP-1 isoforms in inflamed and non-inflamed tonsils.** Frozen sections of normal and inflamed tonsil were stained with JG2.10 (biotinylated) and 2D10, the former detecting only the full-length isoform and the latter detecting both isoforms. 2D10 and JG 2.10 stainings on blood vasculature are seen to co-localize in all three samples. **A**: non-inflamed tonsil, **B**: chronically inflamed tonsils, **C**: acutely inflamed tonsils. Scale bar 100 µm.
(PDF)

Table S1 **Antibodies detecting VAP-1 and VAP-1Δ3.** Listed are the antibodies detecting either one of the two isoforms, and the way they discriminate between the two.
(PDF)

## References

[1] Ley K, Laudanna C, Cybulsky MI, Nourshargh S (2007) Getting to the site of inflammation: the leukocyte adhesion cascade updated. Nat Rev Immunol 7: 678–689. 17717539 10.1038/nri2156

[2] Luster AD, Alon R, von Andrian UH (2005) Immune cell migration in inflammation: present and future therapeutic targets. Nat Immunol 6: 1182–1190. 16369557 10.1038/ni1275

[3] Salmi M, Jalkanen S (1992) A 90-kilodalton endothelial cell molecule mediating lymphocyte binding in humans. Science 257: 1407–1409. 1529341 10.1126/science.1529341

[4] Salmi M, Jalkanen S (1996) Human vascular adhesion protein 1 (VAP-1) is a unique sialoglycoprotein that mediates carbohydrate-dependent binding of lymphocytes to endothelial cells. J Exp Med 183: 569–579. 8627168 10.1084/jem.183.2.569 PMC2192471

[5] Salmi M, Kalimo K, Jalkanen S (1993) Induction and function of vascular adhesion protein-1 at sites of inflammation. J Exp Med 178: 2255–2260. 8245796 10.1084/jem.178.6.2255 PMC2191278

[6] Jaakkola K, Kaunismaki K, Tohka S, Yegutkin G, Vanttinen E, et al (1999) Human vascular adhesion protein-1 in smooth muscle cells. Am J Pathol 155: 1953–1965. 10595925 10.1016/S0002-9440(10)65514-9 PMC1866916

[7] Salmi M, Jalkanen S (2001) VAP-1: an adhesin and an enzyme. Trends Immunol 22: 211–216. 11274927 10.1016/s1471-4906(01)01870-1

[8] Jaakkola K, Nikula T, Holopainen R, Vahasilta T, Matikainen MT, et al (2000) In vivo detection of vascular adhesion protein-1 in experimental inflammation. Am J Pathol 157: 463–471. 10934150 10.1016/S0002-9440(10)64558-0 PMC1850117

[9] Salmi M, Jalkanen S (2005) Cell-surface enzymes in control of leukocyte trafficking. Nat Rev Immunol 5: 760–771. 16200079 10.1038/nri1705

[10] Smith DJ, Salmi M, Bono P, Hellman J, Leu T, et al (1998) Cloning of vascular adhesion protein 1 reveals a novel multifunctional adhesion molecule. J Exp Med 188: 17–27. 9653080 10.1084/jem.188.1.17 PMC2525535

[11] Salmi M, Yegutkin GG, Lehvonen R, Koskinen K, Salminen T, et al (2001) A cell surface amine oxidase directly controls lymphocyte migration. Immunity 14: 265–276. 11290336 10.1016/s1074-7613(01)00108-x

[12] Griendling KK, Sorescu D, Lassegue B, Ushio-Fukai M (2000) Modulation of protein kinase activity and gene expression by reactive oxygen species and their role in vascular physiology and pathophysiology. Arterioscler Thromb Vasc Biol 20: 2175–2183. 11031201 10.1161/01.atv.20.10.2175

[13] Mathys KC, Ponnampalam SN, Padival S, Nagaraj RH (2002) Semicarbazide-sensitive amine oxidase in aortic smooth muscle cells mediates synthesis of a methylglyoxal-AGE: implications for vascular complications in diabetes. Biochem Biophys Res Commun 297: 863–869. 12359232 10.1016/s0006-291x(02)02293-3

[14] Zorzano A, Abella A, Marti L, Carpene C, Palacin M, et al (2003) Semicarbazide-sensitive amine oxidase activity exerts insulin-like effects on glucose metabolism and insulin-signaling pathways in adipose cells. Biochim Biophys Acta 1647: 3–9. 12686100 10.1016/s1570-9639(03)00039-6

[15] Mercier N, Moldes M, El Hadri K, Feve B (2001) Semicarbazide-sensitive amine oxidase activation promotes adipose conversion of 3T3-L1 cells. Biochem J 358: 335–342. 11513731 10.1042/0264-6021:3580335 PMC1222065

[16] Gokturk C, Nilsson J, Nordquist J, Kristensson M, Svensson K, et al (2003) Overexpression of semicarbazide-sensitive amine oxidase in smooth muscle cells leads to an abnormal structure of the aortic elastic laminas. Am J Pathol 163: 1921–1928. 14578191 10.1016/S0002-9440(10)63550-X PMC1892430

[17] Wang ET, Sandberg R, Luo S, Khrebtukova I, Zhang L, et al (2008) Alternative isoform regulation in human tissue transcriptomes. Nature 456: 470–476. 18978772 10.1038/nature07509 PMC2593745

[18] Kalsotra A, Cooper TA (2011) Functional consequences of developmentally regulated alternative splicing. Nat Rev Genet 12: 715–729. 21921927 10.1038/nrg3052 PMC3321218

[19] Kanadia RN, Cepko CL (2010) Alternative splicing produces high levels of noncoding isoforms of bHLH transcription factors during development. Genes Dev 24: 229–234. 20080942 10.1101/gad.1847110 PMC2811824

[20] Sunmonu NA, Li K, Li JY (2011) Numerous isoforms of Fgf8 reflect its multiple roles in the developing brain. J Cell Physiol 226: 1722–1726. 21506104 10.1002/jcp.22587 PMC3071877

[21] Kim E, Goren A, Ast G (2008) Alternative splicing: current perspectives. Bioessays 30: 38–47. 18081010 10.1002/bies.20692

[22] Rehwinkel J, Raes J, Izaurralde E (2006) Nonsense-mediated mRNA decay: Target genes and functional diversification of effectors. Trends Biochem Sci 31: 639–646. 17010613 10.1016/j.tibs.2006.09.005

[23] Shyu AB, Wilkinson MF, van Hoof A (2008) Messenger RNA regulation: to translate or to degrade. Embo J 27: 471–481. 18256698 10.1038/sj.emboj.7601977 PMC2241649

[24] Zhang X, Azhar G, Huang C, Cui C, Zhong Y, et al (2007) Alternative splicing and nonsense-mediated mRNA decay regulate gene expression of serum response factor. Gene 400: 131–139. 17629633 10.1016/j.gene.2007.06.008

[25] Jaffe EA, Nachman RL, Becker CG, Minick CR (1973) Culture of human endothelial cells derived from umbilical veins. Identification by morphologic and immunologic criteria. J Clin Invest 52: 2745–2756. 4355998 10.1172/JCI107470 PMC302542

[26] Jalkanen S, Bargatze RF, de los Toyos J, Butcher EC (1987) Lymphocyte recognition of high endothelium: antibodies to distinct epitopes of an 85–95-kD glycoprotein antigen differentially inhibit lymphocyte binding to lymph node, mucosal, or synovial endothelial cells. J Cell Biol 105: 983–990. 2442176 10.1083/jcb.105.2.983 PMC2114763

[27] Irjala H, Elima K, Johansson EL, Merinen M, Kontula K, et al (2003) The same endothelial receptor controls lymphocyte traffic both in vascular and lymphatic vessels. Eur J Immunol 33: 815–824. 12616502 10.1002/eji.200323859

[28] Hiltunen MO, Laitinen M, Turunen MP, Jeltsch M, Hartikainen J, et al (2000) Intravascular adenovirus-mediated VEGF-C gene transfer reduces neointima formation in balloon-denuded rabbit aorta. Circulation 102: 2262–2268. 11056103 10.1161/01.cir.102.18.2262

[29] Koskinen K, Vainio PJ, Smith DJ, Pihlavisto M, Yla-Herttuala S, et al (2004) Granulocyte transmigration through the endothelium is regulated by the oxidase activity of vascular adhesion protein-1 (VAP-1). Blood 103: 3388–3395. 14726375 10.1182/blood-2003-09-3275

[30] Laitinen M, Makinen K, Manninen H, Matsi P, Kossila M, et al (1998) Adenovirus-mediated gene transfer to lower limb artery of patients with chronic critical leg ischemia. Hum Gene Ther 9: 1481–1486. 9681419 10.1089/hum.1998.9.10-1481

[31] Chirgwin J, Przybyla A, MacDonald R, Rutter W (1979) Isolation of biologically active ribonucleic acid from sources enriched in ribonuclease. Biochemistry 18: 5294–5299. 518835 10.1021/bi00591a005

[32] Junttila TT, Laato M, Vahlberg T, Soderstrom KO, Visakorpi T, et al (2003) Identification of patients with transitional cell carcinoma of the bladder overexpressing ErbB2, ErbB3, or specific ErbB4 isoforms: real-time reverse transcription-PCR analysis in estimation of ErbB receptor status from cancer patients. Clin Cancer Res 9: 5346–5357. 14614020

[33] Kaitaniemi S, Elovaara H, Gron K, Kidron H, Liukkonen J, et al (2009) The unique substrate specificity of human AOC2, a semicarbazide-sensitive amine oxidase. Cell Mol Life Sci 66: 2743–2757. 19588076 10.1007/s00018-009-0076-5 PMC11115939

[34] Tapon N, Nagata K, Lamarche N, Hall A (1998) A new rac target POSH is an SH3-containing scaffold protein involved in the JNK and NF-kappaB signalling pathways. Embo J 17: 1395–1404. 9482736 10.1093/emboj/17.5.1395 PMC1170487

[35] Airenne TT, Nymalm Y, Kidron H, Smith DJ, Pihlavisto M, et al (2005) Crystal structure of the human vascular adhesion protein-1: unique structural features with functional implications. Protein Sci 14: 1964–1974. 16046623 10.1110/ps.051438105 PMC2279308

[36] Ernberg K, McGrath AP, Peat TS, Adams TE, Xiao X, et al (2010) A new crystal form of human vascular adhesion protein 1. Acta Crystallogr Sect F Struct Biol Cryst Commun 66: 1572–1578. 10.1107/S1744309110041515 PMC2998357 21139198

[37] Elovaara H, Kidron H, Parkash V, Nymalm Y, Bligt E, et al (2011) Identification of two imidazole binding sites and key residues for substrate specificity in human primary amine oxidase AOC3. Biochemistry 50: 5507–5520. 21585208 10.1021/bi200117z

[38] Matsunami H, Okajima T, Hirota S, Yamaguchi H, Hori H, et al (2004) Chemical rescue of a site-specific mutant of bacterial copper amine oxidase for generation of the topa quinone cofactor. Biochemistry 43: 2178–2187. 14979714 10.1021/bi0361923

[39] Salmi M, Hellman J, Jalkanen S (1998) The role of two distinct endothelial molecules, vascular adhesion protein-1 and peripheral lymph node addressin, in the binding of lymphocyte subsets to human lymph nodes. J Immunol 160: 5629–5636. 9605169

[40] Conti E, Izaurralde E (2005) Nonsense-mediated mRNA decay: molecular insights and mechanistic variations across species. Curr Opin Cell Biol 17: 316–325. 15901503 10.1016/j.ceb.2005.04.005

[41] Chang YF, Imam JS, Wilkinson MF (2007) The nonsense-mediated decay RNA surveillance pathway. Annu Rev Biochem 76: 51–74. 17352659 10.1146/annurev.biochem.76.050106.093909

[42] Nagy E, Maquat LE (1998) A rule for termination-codon position within intron-containing genes: when nonsense affects RNA abundance. Trends Biochem Sci 23: 198–199. 9644970 10.1016/s0968-0004(98)01208-0

[43] Harada N, Yamada Y, Tsukiyama K, Yamada C, Nakamura Y, et al (2008) A novel GIP receptor splice variant influences GIP sensitivity of pancreatic beta-cells in obese mice. Am J Physiol Endocrinol Metab 294: E61–68. 17971513 10.1152/ajpendo.00358.2007

[44] Sztainberg Y, Kuperman Y, Issler O, Gil S, Vaughan J, et al (2009) A novel corticotropin-releasing factor receptor splice variant exhibits dominant negative activity: a putative link to stress-induced heart disease. Faseb J 23: 2186–2196. 19246489 10.1096/fj.08-128066 PMC2704597

[45] Zarei MM, Zhu N, Alioua A, Eghbali M, Stefani E, et al (2001) A novel MaxiK splice variant exhibits dominant-negative properties for surface expression. J Biol Chem 276: 16232–16239. 11278440 10.1074/jbc.M008852200

[46] Belaguli NS, Zhou W, Trinh TH, Majesky MW, Schwartz RJ (1999) Dominant negative murine serum response factor: alternative splicing within the activation domain inhibits transactivation of serum response factor binding targets. Mol Cell Biol 19: 4582–4591. 10373507 10.1128/mcb.19.7.4582 PMC84256

[47] Davies JQ, Chang GW, Yona S, Gordon S, Stacey M, et al (2007) The role of receptor oligomerization in modulating the expression and function of leukocyte adhesion-G protein-coupled receptors. J Biol Chem 282: 27343–27353. 17620333 10.1074/jbc.M704096200

[48] Lorenz M, Hewing B, Hui J, Zepp A, Baumann G, et al (2007) Alternative splicing in intron 13 of the human eNOS gene: a potential mechanism for regulating eNOS activity. Faseb J 21: 1556–1564. 17264164 10.1096/fj.06-7434com

[49] McElvaine AT, Mayo KE (2006) A dominant-negative human growth hormone-releasing hormone (GHRH) receptor splice variant inhibits GHRH binding. Endocrinology 147: 1884–1894. 16423869 10.1210/en.2005-1488

[50] Gomes AR, Ferreira JS, Paternain AV, Lerma J, Duarte CB, et al (2008) Characterization of alternatively spliced isoforms of AMPA receptor subunits encoding truncated receptors. Mol Cell Neurosci 37: 323–334. 18065236 10.1016/j.mcn.2007.10.008

[51] Kino T, Su YA, Chrousos GP (2009) Human glucocorticoid receptor isoform beta: recent understanding of its potential implications in physiology and pathophysiology. Cell Mol Life Sci 66: 3435–3448. 19633971 10.1007/s00018-009-0098-z PMC2796272

[52] van der Vaart M, Schaaf MJ (2009) Naturally occurring C-terminal splice variants of nuclear receptors. Nucl Recept Signal 7: e007. 19636396 10.1621/nrs.07007 PMC2716050

[53] van Rijn RM, van Marle A, Chazot PL, Langemeijer E, Qin Y, et al (2008) Cloning and characterization of dominant negative splice variants of the human histamine H4 receptor. Biochem J 414: 121–131. 18452403 10.1042/BJ20071583

[54] deLano W (2002) The PyMOL Molecular Graphics System. DeLano Scientific, San Carlos, CA, USA. Available: http://www.pymol.org. Accessed 2012 Dec 21.

