# Supplementary material for: Functional Modulation of Vascular Adhesion Protein-1 by a Novel Splice Variant
Source: PLoS One. 2013 Jan 18;8(1):e54151. doi: 10.1371/journal.pone.0054151 (PMC3548902; doi:10.1371/journal.pone.0054151)
Supplement: Figure S1 — Aligned nucleotide sequences of VAP-1 and VAP-1Δ3. VAP1 represents the full length VAP-1 and VAP1Δ3 the alternatively spliced shorter transcript of VAP-1. The translation start codon (highlighted in yellow) and the stop codons (red) are indicated. (DOC) [file pone.0054151.s001.doc]

Suppl. figure 1

**10 20 30 40 50 60 70 80 90 100**

**vap1 ATGAACCAGAAGACAATCCTCGTGCTCCTCATTCTGGCCGTCATCACCATCTTTGCCTTGGTTTGTGTCCTGCTGGTGGGCAGGGGTGGAGATGGGGGTG**

**::::::::::::::::::::::::::::::::::::::::::::::::::::::::::::::::::::::::::::::::::::::::::::::::::::**

**vap1Δ3 ATGAACCAGAAGACAATCCTCGTGCTCCTCATTCTGGCCGTCATCACCATCTTTGCCTTGGTTTGTGTCCTGCTGGTGGGCAGGGGTGGAGATGGGGGTG**

**140 150 160 170 180 190 200 210 220 230**

**110 120 130 140 150 160 170 180 190 200**

**vap1 AACCCAGCCAGCTTCCCCATTGCCCCTCTGTATCTCCCAGTGCCCAGCCTTGGACACACCCTGGCCAGAGCCAGCTGTTTGCAGACCTGAGCCGAGAGGA**

**::::::::::::::::::::::::::::::::::::::::::::::::::::::::::::::::::::::::::::::::::::::::::::::::::::**

**vap1Δ3 AACCCAGCCAGCTTCCCCATTGCCCCTCTGTATCTCCCAGTGCCCAGCCTTGGACACACCCTGGCCAGAGCCAGCTGTTTGCAGACCTGAGCCGAGAGGA**

**240 250 260 270 280 290 300 310 320 330**

**210 220 230 240 250 260 270 280 290 300**

**vap1 GCTGACGGCTGTGATGCGCTTTCTGACCCAGCGGCTGGGGCCAGGGCTGGTGGATGCAGCCCAGGCCCGGCCCTCGGACAACTGTGTCTTCTCAGTGGAG**

**::::::::::::::::::::::::::::::::::::::::::::::::::::::::::::::::::::::::::::::::::::::::::::::::::::**

**vap1Δ3 GCTGACGGCTGTGATGCGCTTTCTGACCCAGCGGCTGGGGCCAGGGCTGGTGGATGCAGCCCAGGCCCGGCCCTCGGACAACTGTGTCTTCTCAGTGGAG**

**340 350 360 370 380 390 400 410 420 430**

**310 320 330 340 350 360 370 380 390 400**

**vap1 TTGCAGCTGCCTCCCAAGGCTGCAGCCCTGGCTCACTTGGACAGGGGGAGCCCCCCACCTGCCCGGGAGGCACTGGCCATCGTCTTCTTTGGCAGGCAAC**

**::::::::::::::::::::::::::::::::::::::::::::::::::::::::::::::::::::::::::::::::::::::::::::::::::::**

**vap1Δ3 TTGCAGCTGCCTCCCAAGGCTGCAGCCCTGGCTCACTTGGACAGGGGGAGCCCCCCACCTGCCCGGGAGGCACTGGCCATCGTCTTCTTTGGCAGGCAAC**

**440 450 460 470 480 490 500 510 520 530**

**410 420 430 440 450 460 470 480 490 500**

**vap1 CCCAGCCCAACGTGAGTGAGCTGGTGGTGGGGCCACTGCCTCACCCCTCCTACATGCGGGACGTGACTGTGGAGCGTCATGGAGGCCCCCTGCCCTATCA**

**::::::::::::::::::::::::::::::::::::::::::::::::::::::::::::::::::::::::::::::::::::::::::::::::::::**

**vap1Δ3 CCCAGCCCAACGTGAGTGAGCTGGTGGTGGGGCCACTGCCTCACCCCTCCTACATGCGGGACGTGACTGTGGAGCGTCATGGAGGCCCCCTGCCCTATCA**

**540 550 560 570 580 590 600 610 620 630**

**510 520 530 540 550 560 570 580 590 600**

**vap1 CCGACGCCCCGTGCTGTTCCAAGAGTACCTGGACATAGACCAGATGATCTTCAACAGAGAGCTGCCCCAGGCTTCTGGGCTTCTCCACCACTGTTGCTTC**

**::::::::::::::::::::::::::::::::::::::::::::::::::::::::::::::::::::::::::::::::::::::::::::::::::::**

**vap1Δ3 CCGACGCCCCGTGCTGTTCCAAGAGTACCTGGACATAGACCAGATGATCTTCAACAGAGAGCTGCCCCAGGCTTCTGGGCTTCTCCACCACTGTTGCTTC**

**640 650 660 670 680 690 700 710 720 730**

**610 620 630 640 650 660 670 680 690 700**

**vap1 TACAAGCACCGGGGACGGAACCTGGTGACAATGACCACGGCTCCCCGTGGTCTGCAATCAGGGGACCGGGCCACCTGGTTTGGCCTCTACTACAACATCT**

**::::::::::::::::::::::::::::::::::::::::::::::::::::::::::::::::::::::::::::::::::::::::::::::::::::**

**vap1Δ3 TACAAGCACCGGGGACGGAACCTGGTGACAATGACCACGGCTCCCCGTGGTCTGCAATCAGGGGACCGGGCCACCTGGTTTGGCCTCTACTACAACATCT**

**740 750 760 770 780 790 800 810 820 830**

**710 720 730 740 750 760 770 780 790 800**

**vap1 CGGGCGCTGGGTTCTTCCTGCACCACGTGGGCTTGGAGCTGCTAGTGAACCACAAGGCCCTTGACCCTGCCCGCTGGACTATCCAGAAGGTGTTCTATCA**

**::::::::::::::::::::::::::::::::::::::::::::::::::::::::::::::::::::::::::::::::::::::::::::::::::::**

**vap1Δ3 CGGGCGCTGGGTTCTTCCTGCACCACGTGGGCTTGGAGCTGCTAGTGAACCACAAGGCCCTTGACCCTGCCCGCTGGACTATCCAGAAGGTGTTCTATCA**

**840 850 860 870 880 890 900 910 920 930**

**810 820 830 840 850 860 870 880 890 900**

**vap1 AGGCCGCTACTACGACAGCCTGGCCCAGCTGGAGGCCCAGTTTGAGGCCGGCCTGGTGAATGTGGTGCTGATCCCAGACAATGGCACAGGTGGGTCCTGG**

**::::::::::::::::::::::::::::::::::::::::::::::::::::::::::::::::::::::::::::::::::::::::::::::::::::**

**vap1Δ3 AGGCCGCTACTACGACAGCCTGGCCCAGCTGGAGGCCCAGTTTGAGGCCGGCCTGGTGAATGTGGTGCTGATCCCAGACAATGGCACAGGTGGGTCCTGG**

**940 950 960 970 980 990 1000 1010 1020 1030**

**910 920 930 940 950 960 970 980 990 1000**

**vap1 TCCCTGAAGTCCCCTGTGCCCCCGGGTCCAGCTCCCCCTCTACAGTTCTATCCCCAAGGCCCCCGCTTCAGTGTCCAGGGAAGTCGAGTGGCCTCCTCAC**

**::::::::::::::::::::::::::::::::::::::::::::::::::::::::::::::::::::::::::::::::::::::::::::::::::::**

**vap1Δ3 TCCCTGAAGTCCCCTGTGCCCCCGGGTCCAGCTCCCCCTCTACAGTTCTATCCCCAAGGCCCCCGCTTCAGTGTCCAGGGAAGTCGAGTGGCCTCCTCAC**

**1040 1050 1060 1070 1080 1090 1100 1110 1120 1130**

**1010 1020 1030 1040 1050 1060 1070 1080 1090 1100**

**vap1 TGTGGACTTTCTCCTTTGGCCTCGGAGCATTCAGTGGCCCAAGGATCTTTGACGTTCGCTTCCAAGGAGAAAGACTAGTTTATGAGATAAGCCTCCAAGA**

**::::::::::::::::::::::::::::::::::::::::::::::::::::::::::::::::::::::::::::::::::::::::::::::::::::**

**vap1Δ3 TGTGGACTTTCTCCTTTGGCCTCGGAGCATTCAGTGGCCCAAGGATCTTTGACGTTCGCTTCCAAGGAGAAAGACTAGTTTATGAGATAAGCCTCCAAGA**

**1140 1150 1160 1170 1180 1190 1200 1210 1220 1230**

**1110 1120 1130 1140 1150 1160 1170 1180 1190 1200**

**vap1 GGCCTTGGCCATCTATGGTGGAAATTCCCCAGCAGCAATGACGACCCGCTATGTGGATGGAGGCTTTGGCATGGGCAAGTACACCACGCCCCTGACCCGT**

**::::::::::::::::::::::::::::::::::::::::::::::::::::::::::::::::::::::::::::::::::::::::::::::::::::**

**vap1Δ3 GGCCTTGGCCATCTATGGTGGAAATTCCCCAGCAGCAATGACGACCCGCTATGTGGATGGAGGCTTTGGCATGGGCAAGTACACCACGCCCCTGACCCGT**

**1240 1250 1260 1270 1280 1290 1300 1310 1320 1330**

**1210 1220 1230 1240 1250 1260 1270 1280 1290 1300**

**vap1 GGGGTGGACTGCCCCTACTTGGCCACCTACGTGGACTGGCACTTCCTTTTGGAGTCCCAGGCCCCCAAGACAATACGTGATGCCTTTTGTGTGTTTGAAC**

**::::::::::::::::::::::::::::::::::::::::::::::::::::::::::::::::::::::::::::::::::::::::::::::::::::**

**vap1Δ3 GGGGTGGACTGCCCCTACTTGGCCACCTACGTGGACTGGCACTTCCTTTTGGAGTCCCAGGCCCCCAAGACAATACGTGATGCCTTTTGTGTGTTTGAAC**

**1340 1350 1360 1370 1380 1390 1400 1410 1420 1430**

**1310 1320 1330 1340 1350 1360 1370 1380 1390 1400**

**vap1 AGAACCAGGGCCTCCCCCTGCGGCGACACCACTCAGATCTCTACTCGCACTACTTTGGGGGTCTTGCGGAAACGGTGCTGGTCGTCAGATCTATGTCCAC**

**::::::::::::::::::::::::::::::::::::::::::::::::::::::::::::::::::::::::::::::::::::::::::::::::::::**

**vap1Δ3 AGAACCAGGGCCTCCCCCTGCGGCGACACCACTCAGATCTCTACTCGCACTACTTTGGGGGTCTTGCGGAAACGGTGCTGGTCGTCAGATCTATGTCCAC**

**1440 1450 1460 1470 1480 1490 1500 1510 1520 1530**

**1410 1420 1430 1440 1450 1460 1470 1480 1490 1500**

**vap1 CTTGCTCAACTATGACTATGTGTGGGATACGGTCTTCCACCCCAGTGGGGCCATAGAAATACGATTCTATGCCACGGGCTACATCAGCTCGGCATTCCTC**

**::::::::::::::::::::::::::::::::::::::::::::::::::::::::::::::::::::::::::::::::::::::::::::::::::::**

**vap1Δ3 CTTGCTCAACTATGACTATGTGTGGGATACGGTCTTCCACCCCAGTGGGGCCATAGAAATACGATTCTATGCCACGGGCTACATCAGCTCGGCATTCCTC**

**1540 1550 1560 1570 1580 1590 1600 1610 1620 1630**

**1510 1520 1530 1540 1550 1560 1570 1580 1590 1600**

**vap1 TTTGGTGCTACTGGGAAGTACGGGAACCAAGTGTCAGAGCACACCCTGGGCACGGTCCACACCCACAGCGCCCACTTCAAGGTGGATCTGGATGTAGCAG**

**::::::::::::::::::::::::::::::::::::::::::::::::::::::::::::::::::::::::::::::::::::::::::::::::::::**

**vap1Δ3 TTTGGTGCTACTGGGAAGTACGGGAACCAAGTGTCAGAGCACACCCTGGGCACGGTCCACACCCACAGCGCCCACTTCAAGGTGGATCTGGATGTAGCAG**

**1640 1650 1660 1670 1680 1690 1700 1710 1720 1730**

**1610 1620 1630 1640 1650 1660 1670 1680 1690 1700**

**vap1 GACTGGAGAACTGGGTCTGGGCCGAGGATATGGTCTTTGTCCCCATGGCTGTGCCCTGGAGCCCTGAGCACCAGCTGCAGAGGCTGCAGGTGACCCGGAA**

**::::::::::::::::::::::::::::::::::::::::::::::::::::::::::::::::::::::::::::::::::::::::::::::::::::**

**vap1Δ3 GACTGGAGAACTGGGTCTGGGCCGAGGATATGGTCTTTGTCCCCATGGCTGTGCCCTGGAGCCCTGAGCACCAGCTGCAGAGGCTGCAGGTGACCCGGAA**

**1740 1750 1760 1770 1780 1790 1800 1810 1820 1830**

**1710 1720 1730 1740 1750 1760 1770 1780 1790 1800**

**vap1 GCTGCTGGAGATGGAGGAGCAGGCCGCCTTCCTCGTGGGAAGCGCCACCCCTCGCTACCTGTACCTGGCCAGCAACCACAGCAACAAGTGGGGTCACCCC**

**::::::::::::::::::::::::::::::::::::::::::::::::::::::::::::::::::::::::::::::::::::::::::::::::::::**

**vap1Δ3 GCTGCTGGAGATGGAGGAGCAGGCCGCCTTCCTCGTGGGAAGCGCCACCCCTCGCTACCTGTACCTGGCCAGCAACCACAGCAACAAGTGGGGTCACCCC**

**1840 1850 1860 1870 1880 1890 1900 1910 1920 1930**

**1810 1820 1830 1840 1850 1860 1870 1880 1890 1900**

**vap1 CGGGGCTACCGCATCCAGATGCTCAGCTTTGCTGGAGAGCCGCTGCCCCAAAACAGCTCCATGGCGAGAGGCTTCAGCTGGGAGAGGTACCAGCTGGCTG**

**::::::::::::::::::::::::::::::::::::::::::::::::::::::::::::::::::::::::::::::::::::::**

**vap1Δ3 CGGGGCTACCGCATCCAGATGCTCAGCTTTGCTGGAGAGCCGCTGCCCCAAAACAGCTCCATGGCGAGAGGCTTCAGCTGGGAGAG--------------**

**1940 1950 1960 1970 1980 1990 2000 2010 2020**

**1910 1920 1930 1940 1950 1960 1970 1980 1990 2000**

**vap1 TGACCCAGCGGAAGGAGGAGGAGCCCAGTAGCAGCAGCGTTTTCAATCAGAATGACCCTTGGGCCCCCACTGTGGATTTCAGTGACTTCATCAACAATGA**

**vap1Δ3 ----------------------------------------------------------------------------------------------------**

**2010 2020 2030 2040 2050 2060 2070 2080 2090 2100**

**vap1 GACCATTGCTGGAAAGGATTTGGTGGCCTGGGTGACAGCTGGTTTTCTGCATATCCCACATGCAGAGGACATTCCTAACACAGTGACTGTGGGGAACGGC**

**::::::::::::::::::::::::::::::::::::::::::::::::::::::::::::::::::::::::::::::::::::**

**vap1Δ3 ----------------GATTTGGTGGCCTGGGTGACAGCTGGTTTTCTGCATATCCCACATGCAGAGGACATTCCTAACACAGTGACTGTGGGGAACGGC**

**2030 2040 2050 2060 2070 2080 2090 2100**

**2110 2120 2130 2140 2150 2160 2170 2180 2190 2200**

**vap1 GTGGGCTTCTTCCTCCGACCCTATAACTTCTTTGACGAAGACCCCTCCTTCTACTCTGCCGACTCCATCTACTTCCGAGGGGACCAGGATGCTGGGGCCT**

**::::::::::::::::::::::::::::::::::::::::::::::::::::::::::::::::::::::::::::::::::::::::::::::::::::**

**vap1Δ3 GTGGGCTTCTTCCTCCGACCCTATAACTTCTTTGACGAAGACCCCTCCTTCTACTCTGCCGACTCCATCTACTTCCGAGGGGACCAGGATGCTGGGGCCT**

**2110 2120 2130 2140 2150 2160 2170 2180 2190 2200**

**2210 2220 2230 2240 2250 2260 2270 2280 2290 2300**

**vap1 GCGAGGTCAACCCCCTAGCTTGCCTGCCCCAGGCTGCTGCCTGTGCCCCCGACCTCCCTGCCTTCTCCCACGGGGGCTTCTCTCACAACTAGGCGGTCCT**

**::::::::::::::::::::::::::::::::::::::::::::::::::::::::::::::::::::::::::::::::::::::::::::::::::::**

**vap1Δ3 GCGAGGTCAACCCCCTAGCTTGCCTGCCCCAGGCTGCTGCCTGTGCCCCCGACCTCCCTGCCTTCTCCCACGGGGGCTTCTCTCACAACTAGGCGGTCCT**

**2210 2220 2230 2240 2250 2260 2270 2280 2290 2300**

**2310 2320 2330 2340 2350 2360 2370 2380 2390 2400**

**vap1 GGGATGGGGCATGTGGCCAAGGGCTCCAGGGCCAGGGTGTGAGGGATGGGGAGCAGCTGGGCACTGGGCCGGCAGCCTGGTTCCCTCTTTCCTGTGCCAG**

**::::::::::::::::::::::::::::::::::::::::::::::::::::::::::::::::::::::::::::::::::::::::::::::::::::**

**vap1Δ3 GGGATGGGGCATGTGGCCAAGGGCTCCAGGGCCAGGGTGTGAGGGATGGGGAGCAGCTGGGCACTGGGCCGGCAGCCTGGTTCCCTCTTTCCTGTGCCAG**

**2310 2320 2330 2340 2350 2360 2370 2380 2390 2400**

**2410 2420**

**vap1 GACTCTCTTTCTTCCACTACC**

**:::::::::::::::::::::**

**vap1Δ3 GACTCTCTTTCTTCCACTACC**

**2410 2420**
