# Supplementary figures and images for: Functional Modulation of Vascular Adhesion Protein-1 by a Novel Splice Variant
Source: PLoS One. 2013 Jan 18;8(1):e54151. doi: 10.1371/journal.pone.0054151 (PMC3548902; doi:10.1371/journal.pone.0054151)

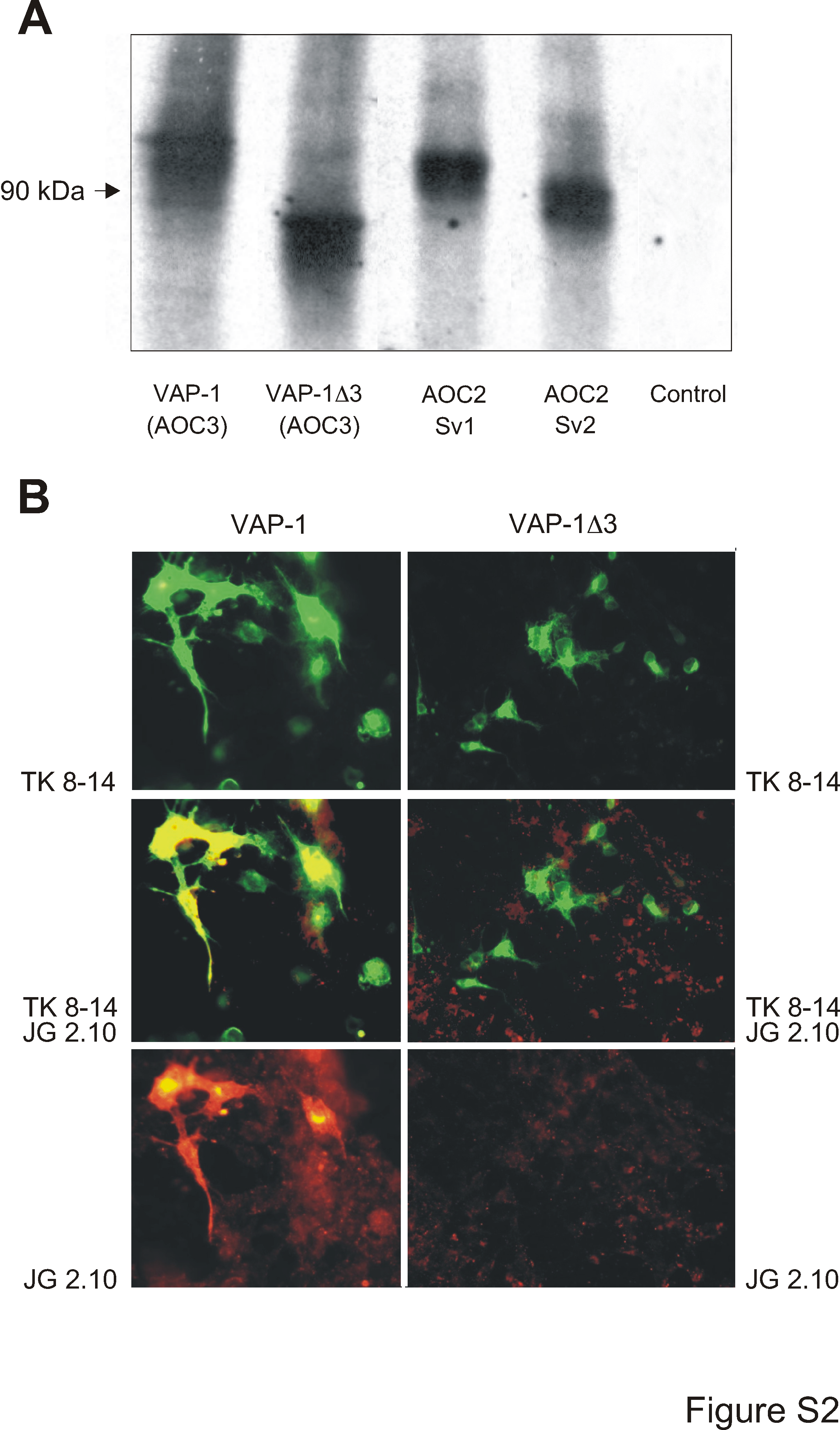

Supplement: Figure S2 — In vitro transcription/translation and immunostainings of the two VAP-1 splice variants. (A) In vitro transcription/translation reactions were performed using the two VAP-1 splice variant cDNAs or the empty vector (control) as templates. For additional size controls, the two AOC2 splice variant clones described before in [33] were also used as templates. The resulting polypeptides were immunoprecipitated with a polyclonal VAP-antibody and run in SDS-PAGE. The size of the monomeric VAP-1 chain (90 kDa) is indicated. (B) HEK293 cells transfected with either the full-length VAP-1 or with VAP-1Δ3 and stained with TK 8–14 and JG 2.10. (TIF) [file pone.0054151.s002.tif]

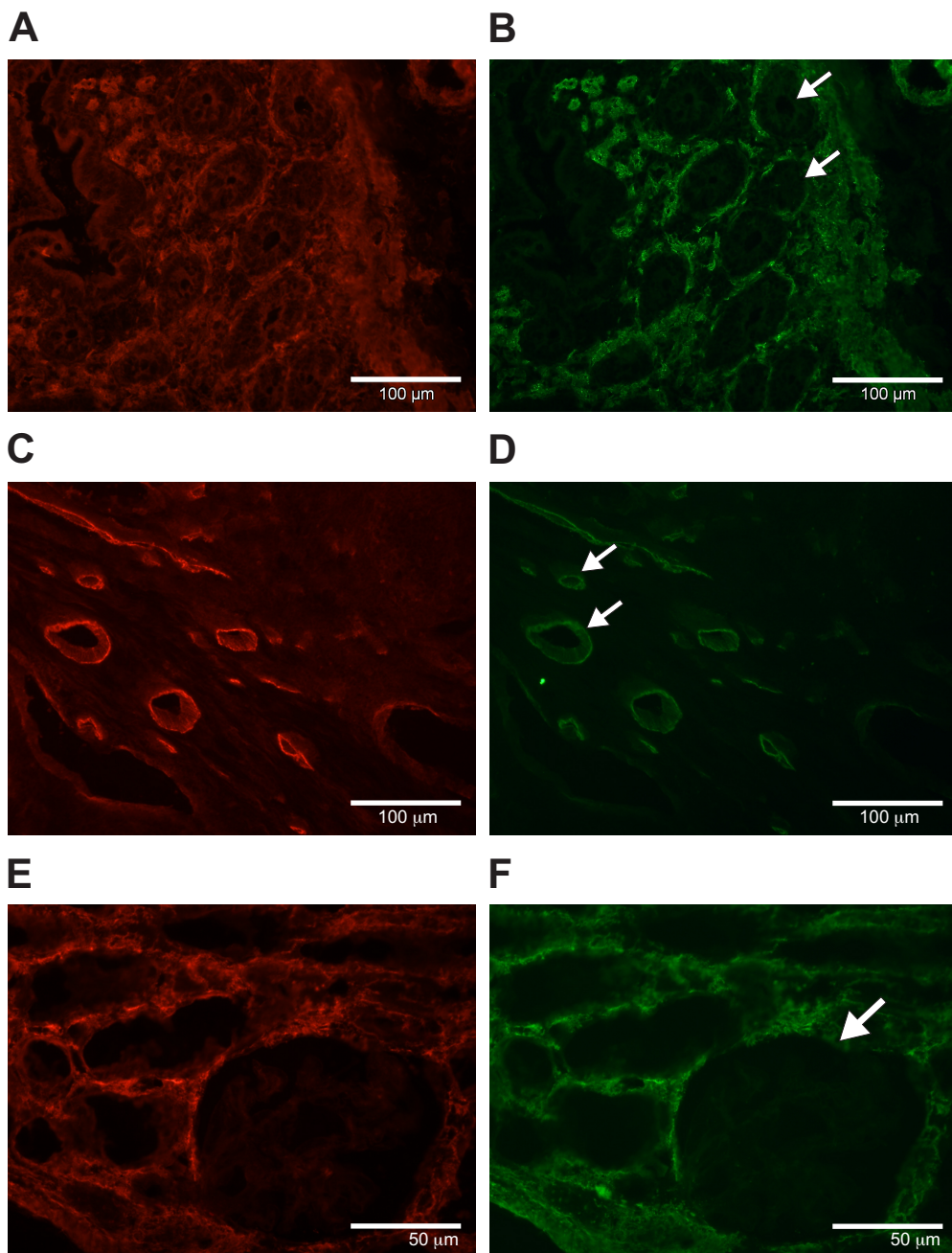

Figure S3

Supplement: Figure S3 — Expression of VAP-1 isoforms in human tissues. Frozen sections of gut (A), tonsil (C), and kidney (E) were stained with JG 2.10, which detects only the full-length isoform of VAP-1. (B, D, F) The corresponding tissues stained with 2D10 which detects both VAP-1 isoforms. In all, the typical VAP-1 staining pattern in the blood vessels can be seen. In B, intestinal villi, in D blood vessels, and in F a glomerulus, are pointed out by arrows. (PDF) [file pone.0054151.s003.pdf]

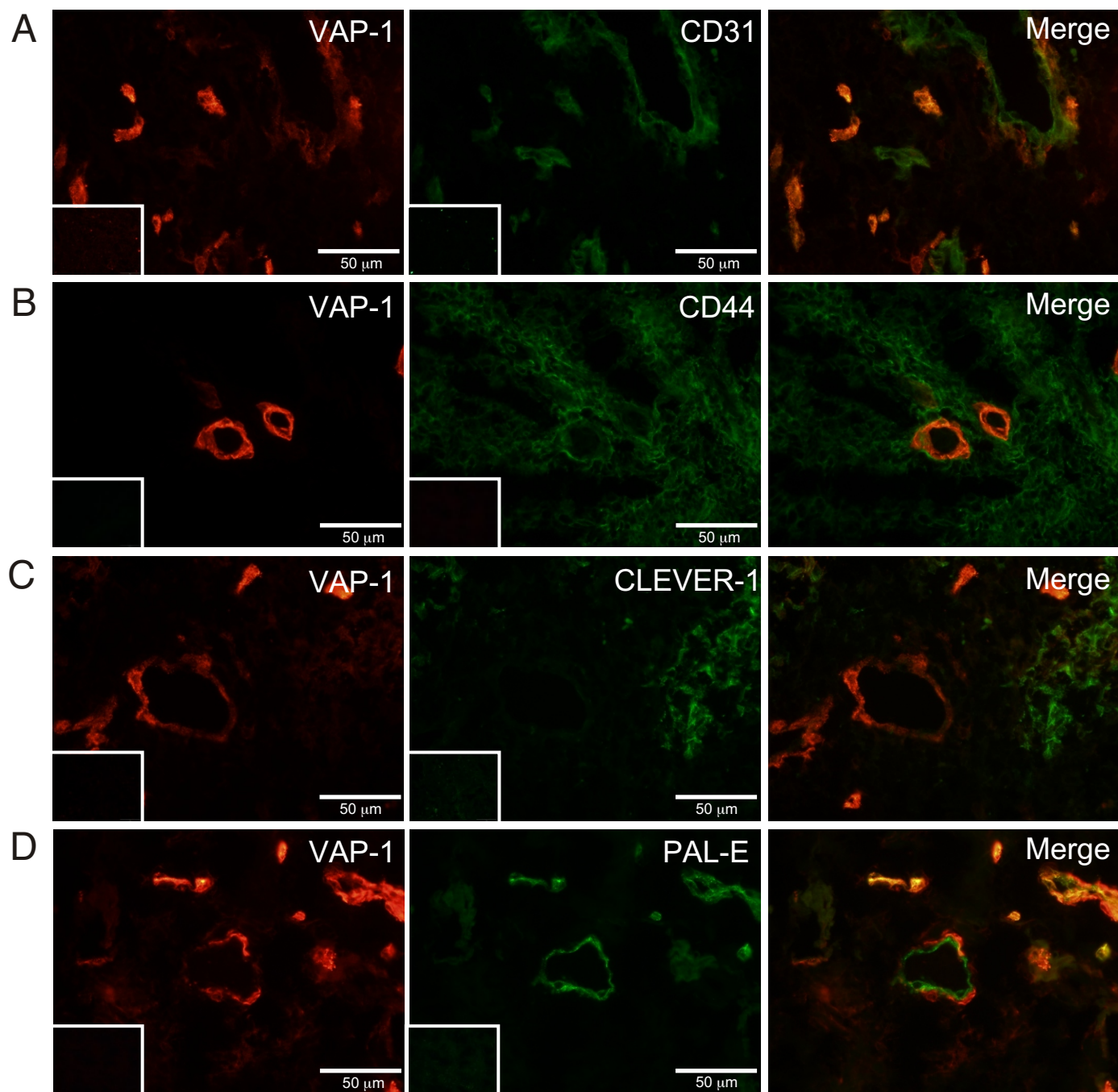

Figure S4

Supplement: Figure S4 — Expression of VAP-1 and various cell type markers in human tissues. Frozen sections of peripheral lymph nodes (A and C) and tonsils (B and D) were stained with JG 2.10, which detects VAP-1, and with other antibodies recognizing particular cell type markers to verify the localization of VAP-1. The markers used are: CD31 and PAL-E for vascular endothelium, CD44 as a pan-cellular marker (excluding high endothelial venules), and CLEVER-1 for lymphatic endothelium. In A, the co-localization of CD31 and VAP-1, and in D, the co-localization of PAL-E and VAP-1 has been shown on blood vasculature. In C and D, VAP-1 is seen not to co-localize with CD44 or CLEVER-1. The inserts in the lower left hand corner depict staining with the negative control antibody. Scale bar 50 µm. (PDF) [file pone.0054151.s004.pdf]

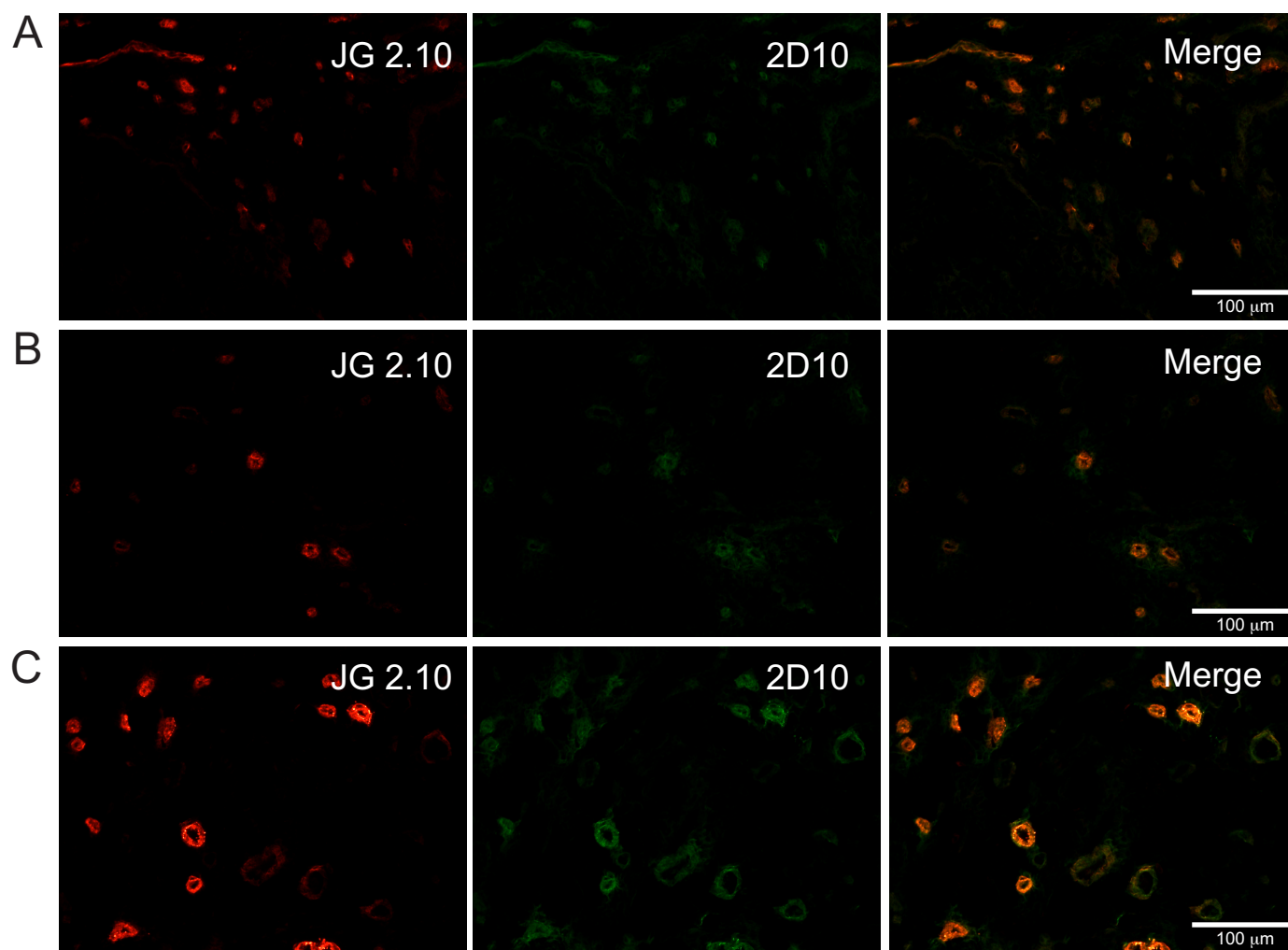

Figure S5

Supplement: Figure S5 — Expression of VAP-1 isoforms in inflamed and non-inflamed tonsils. Frozen sections of normal and inflamed tonsil were stained with JG2.10 (biotinylated) and 2D10, the former detecting only the full-length isoform and the latter detecting both isoforms. 2D10 and JG 2.10 stainings on blood vasculature are seen to co-localize in all three samples. A: non-inflamed tonsil, B: chronically inflamed tonsils, C: acutely inflamed tonsils. Scale bar 100 µm. (PDF) [file pone.0054151.s005.pdf]
