# Supplementary material for: Functional Modulation of Vascular Adhesion Protein-1 by a Novel Splice Variant
Source: PLoS One. 2013 Jan 18;8(1):e54151. doi: 10.1371/journal.pone.0054151 (PMC3548902; doi:10.1371/journal.pone.0054151)
Supplement: Table S1 — Antibodies detecting VAP-1 and VAP-1Δ3. Listed are the antibodies detecting either one of the two isoforms, and the way they discriminate between the two. (PDF) [file pone.0054151.s006.pdf]

Table S1

Antibody	Isotype	Reference	VAP-1	VAP-1Δ3
poly-VAP	rabbit IgG	[S1]	+	+
TK 8-18	mouse IgG_{2a}	[S2]	+	+
TK 8-14	mouse IgG_{2a}	[S2]	+	+
2D10	mouse IgG_1	[S2]	+	+
JG 2.10	rat IgG	[S3]	+	-

S1. Maula SM, Salminen T, Kaitaniemi S, Nymalm Y, Smith DJ, et al. (2005) Carbohydrates located on the top of the "cap" contribute to the adhesive and enzymatic functions of vascular adhesion protein-1. Eur J Immunol 35: 2718-2727.
S2. Kurkijarvi R, Adams DH, Leino R, Mottonen T, Jalkanen S, et al. (1998) Circulating form of human vascular adhesion protein-1 (VAP-1): increased serum levels in inflammatory liver diseases. J Immunol 161: 1549-1557.
S3. Salmi M, Smith DJ, Bono P, Jalkanen S. (1997) Vascular adhesion protein 1. In: Kishimoto T, Hitoshi K, von dem Borne AE, Goyert S, Mason D, et al., editors. Leukocyte typing VI. White cell differentiation antigens. Garland Publishing, Inc. pp. 772-773.
